# Capacity estimates for optical transmission based on the nonlinear Fourier transform

**DOI:** 10.1038/ncomms12710

**Published:** 2016-09-09

**Authors:** Stanislav A. Derevyanko, Jaroslaw E. Prilepsky, Sergei K. Turitsyn

**Affiliations:** 1 Department of Electrical and Computer Engineering, Ben-Gurion University of the Negev, Beer Sheva 84105, Israel; 2 Aston Institute of Photonic Technologies, Aston University, Birmingham B4 7ET, UK

## Abstract

What is the maximum rate at which information can be transmitted error-free in fibre–optic communication systems? For linear channels, this was established in classic works of Nyquist and Shannon. However, despite the immense practical importance of fibre–optic communications providing for >99% of global data traffic, the channel capacity of optical links remains unknown due to the complexity introduced by fibre nonlinearity. Recently, there has been a flurry of studies examining an expected cap that nonlinearity puts on the information-carrying capacity of fibre–optic systems. Mastering the nonlinear channels requires paradigm shift from current modulation, coding and transmission techniques originally developed for linear communication systems. Here we demonstrate that using the integrability of the master model and the nonlinear Fourier transform, the lower bound on the capacity per symbol can be estimated as 10.7 bits per symbol with 500 GHz bandwidth over 2,000 km.

## Abstract (web summary)

Optical fibres enable high-speed communication over long distances, but traditional systems are limited by nonlinear optical effects. Here, the authors quantify the increase in capacity that is made possible by using an alternative approach that uses a nonlinear Fourier transform.

## Graphical abstract

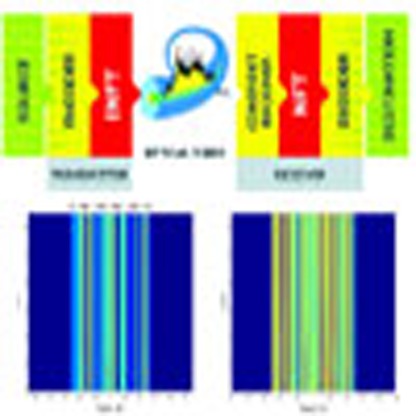

It is hard to overestimate the impact that optical fibre transmission systems have had on everyday life in the ‘information society' era. Although these systems have undergone a long process of increasing engineering complexity and sophistication^1^, the key physical effects that affect system performance remain much the same as before^1^,^2^,^3^,^4^,^5^,^6^,^7^,^8^. These are: chromatic dispersion, fibre Kerr nonlinearity and optical noise. Most of the current optical networks exploit methodologies that were originally developed for linear channels. Thus, it is not surprising that nonlinearity has a detrimental impact on such systems^3^,^4^,^5^,^6^,^7^,^8^, since the only role that it can play within the ‘linear communications' is to serve as a source of signal distortion; examples of the beneficial impact of nonlinearity are relatively scarce^9^,^10^,^11^. It has been predicted that, within the next decade, the existing optical fibre technology will approach the ‘nonlinear transmission limit' (an infamous capacity crunch problem^8^), which caps the achievable rate of error-free data transmission^3^,^4^,^5^,^6^,^8^ (with the first capacity limit estimates taking into account both noise and nonlinearity attributed to the work of Splett *et al*.^12^). Thus, to ‘unlock' the capacity of nonlinear channels, it is necessary to shift the relevant information and communications technology paradigm by introducing truly nonlinear transmission and signal processing techniques. In this work, we adapt techniques developed in nonlinear science to optical communications and use these principally new tools to determine an estimate for the lower bound on nonlinear channel capacity.

The ubiquitous master model governing signal propagation in fibre–optic links is the nonlinear Schrödinger equation (NLSE)^1^,^2^,^5^. The NLSE belongs to the unique class of integrable equations that can be solved via the inverse scattering transform^13^. The latter is an extension of the Fourier transform onto nonlinear systems and is often called the nonlinear Fourier transform (NFT)^14^,^15^. This term indicates that the basic principle of how NFT works is the same as in the linear case: similar to reducing the effect of chromatic dispersion in a linear propagation to a phase rotation in frequency space through the Fourier transform, the NFT transforms the effects of both nonlinearity and dispersion into a trivial linear evolution of the nonlinear spectral data. Therefore, it stands to reason that truly nonlinear techniques of the chromatic dispersion and fibre nonlinearity compensation should rely on NFT-based algorithms in place of linear counterparts. In 1993, Hasegawa and Nyu proposed using discrete eigenvalues (corresponding to solitons) emerging from the NFT to encode and transmit information, as these are not affected by dispersion and nonlinearity^10^,^16^. They termed this approach ‘eigenvalue communications'. Later, Yousefi and Kschischang^17^ used NFT for nonlinear signal multiplexing in multi-user channels. The objective of their approach was to solve the problem of nonlinear crosstalk that occurs in wavelength–division–multiplexed systems. Both ideas have received various generalizations and extensions, and some first experimental implementations have already been reported (see below). In this paper, we refer to both approaches by the umbrella term of NFT.

The existing optical transmission methods employing NFT can be categorized into two general groups. The first one^18^,^19^ employs NFT as an efficient tool for solving NLSE backwards, in a manner similar to digital back propagation^20^. The second approach implies the use of nonlinear modes themselves for the data encoding and transmission^17^,^21^,^22^,^23^,^24^,^25^,^26^. The first consideration of the multiplexing in the nonlinear Fourier domain was presented in ref. 17. We note the recent experiments of Osaka group^27^,^28^, Bülow *et al*.^29^,^30^ and Dong *et al*.^26^, demonstrating the feasibility of the NFT-based optical transmission. Furthermore, the current NFT-based approaches can be classified according to what part of the nonlinear spectrum is used for modulation. The authors of (refs 26, 27, 28, 31, 32) exploited discrete spectra. The novel concept of using the continuous nonlinear spectrum as information carrier was put forward in refs 17, 21, 22, 23, 33. In particular, a method of nonlinear inverse synthesis (NIS) was proposed in refs 21, 22, 23: its purpose is to generate the time domain waveforms starting from a continuous nonlinear spectrum that exactly matches the linear spectrum of the data to be transmitted.

In the following, we address the fundamental question as to whether the achievable information capacity of fibre channels can be enhanced using NFT. In this work, we show that the use of NFT/NIS methods makes it possible to favourably estimate the lower bound of the capacity per symbol for the long-haul fibre networks in the multichannel/multicarrier environment, compared with the conventional modulation techniques. We demonstrate that in a wide range of input power levels, the well-established results from the NLSE perturbation theory^34^,^35^ can be used to formulate an asymptotic channel model in the NFT domain. Using very conservative estimates for the lower bound of capacity^3^,^36^, we derive the estimates for the lower bound for the capacity per symbol of NIS-based transmission (within an approximate model), predicting the lower-bound values of ∼11 (bits/symbol) for 5 × 100 GHz wavelength-division-multiplexing (WDM) Nyquist and orthogonal frequency division multiplexing (OFDM) transmission at 2,000 km. This bound improves logarithmically with the channel bandwidth or subcarrier spacing, see equation (12). Our results also reveal an improvement over the achievable information rates reported recently^37^,^38^, although our goal here is rather to show to the wider community the potential benefits of using the NFT. We also demonstrate that even in the presence of the small inline noise the channel remains free from the nonlinear crosstalk that is thought to be one of the main sources of the spectral efficiency degradation^3^,^4^,^5^,^8^. Since the capacity estimates used to derive these bounds are known to be loose for nonlinear and non-Gaussian information channels^39^, the actual value of the achievable capacity is anticipated to be higher.

## Results

### Model description and basics of NFT and NIS method

The common channel model for optical communications inside a single-mode fibre is the NLSE written for the electrical field envelope *q*(*z*,*t*), perturbed by additive white Gaussian noise (AWGN)^1^,^2^,^4^,^5^. We will mostly work in standard dimensionless units (Supplementary Note 1), and consider the most practically useful case of anomalous dispersion:


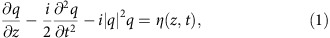


with *z* being a normalized distance along the fibre, *t* is time in frame co-moving with the envelope and the circularly-symmetric AWGN term *η* (having zero mean) is completely characterized by the spectral power density of noise *D* defined via the autocorrelation function: 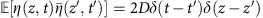, where the overbar means complex conjugate and 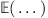 is the expectation value. Such a form of the optical channel corresponds to the amplification scheme, in which the distributed Raman gain exactly compensates for the intrinsic fibre loss^4^,^5^. Traditional (linear) modulation techniques work in time or linear frequency domain, where the evaluation of the maximum achievable error-free transmission rate of channel (1) in symbols per second—that is, the Shannon capacity^40^,^41^—is quite a nontrivial and challenging task^42^. We address the same problem in our work, but specifically for the NFT-based transmission.

The details of the NFT for the NLSE can be found in a great number of works on the subject^10^,^13^,^14^,^15^,^17^. Performing the direct NFT on a pulse *q*(*t*) amounts to solving the so-called Zakharov–Shabat problem, written for two auxiliary functions *v*_1,2_(*t*):


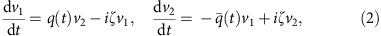


where the input pulse shape *q*(*t*) acts as a potential. Here *ζ* is a (generally complex) eigenvalue, *ζ*=*ξ*+*iρ*, and *q*(*t*) decays as *t*→±∞. To define scattering data (the analogue of Fourier spectrum), for real *ζ*=*ξ*, one selects a specific solution of equation (2), Φ(*t*,*ξ*)=[*φ*_1_,*φ*_2_]^*T*^, by the ‘initial condition' at the trailing end of the pulse: Φ|_*t*→−∞_=[*e*^−*iξt*^,0]^*T*^. Then, the solution at the leading end must necessarily take the form Φ_*t*→+∞_=[*a*(*ξ*)*e*^−*iξt*^,*b*(*ξ*)*e*^*iξt*^]^*T*^, where the functions *a*(*ξ*) and *b*(*ξ*) are called scattering coefficients. The continuous part of the nonlinear spectrum is defined by the ratio named a reflection coefficient: *r*(*ξ*)=*b*(*ξ*)/*a*(*ξ*), and the discrete complex eigenvalues, *ζ*_*n*_, are the zeros of the coefficient *a*(*ξ*) analytically extended into the upper half plane of *ζ*. The forward NFT operation corresponds to mapping of the initial field, *q*(0,*t*), onto a set of scattering data: 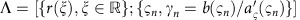, where the index *n* runs over all discrete eigenvalues of Zakharov–Shabat problem.

**Figure 1:**
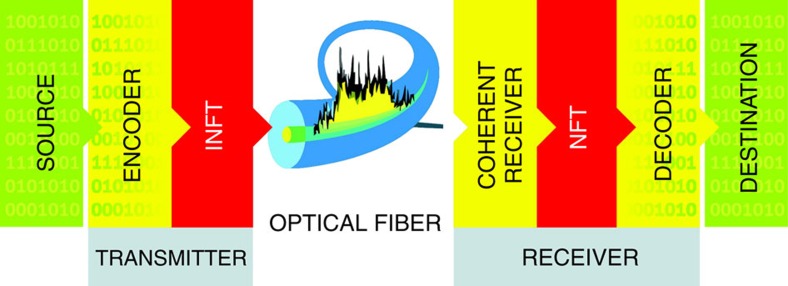
Simplified flowchart of the tranceiver scheme. Shown are: the NFT-based synthesis operation at the transmitter and NFT-based demodulation at the receiver (both transmitter and receiver can also include an FT operation depending on the explicit processing algorithm). For a more detailed block-diagram and a thorough description of the NIS-based optical communication system, see (refs 22, 23).

Figure 1 depicts the simplified flowchart of operations for the NIS NFT-based transmission scheme, see also^21^,^22^,^23^,^24^, and ref. 25 for the experimental set-up scheme. Within the NIS, the parameters of nonlinear modes serve as elementary information carriers, and at the detector one retrieves the data encoded directly from the nonlinear spectrum using the NFT operation. The main advantages of the NIS again the other NFT-based counterparts are as follows. First, insofar as the continuous nonlinear spectrum of our signal matches the linear spectrum of data to be transmitted, the ‘learning curve' for system designers is not very steep, as one can avoid dealing with ‘non-traditional' encoding schemes. Second, the transmission looks very similar to that through a linear dispersive channel. Third, for the continuous spectrum, one can immediately take advantage of the existing efficient modulation formats and adapt those directly for nonlinear spectral communications. In addition, this scheme has been shown to provide higher noise tolerance and the potential for lower numerical complexity than in the case of digital back propagation, outperforming linear compensation in terms of transmission quality^21^,^22^,^23^. Thereby, we will use the NIS as our scheme of choice when providing the capacity estimates of the nonlinear fibre channel, though our approach can be generalized to various other NFT systems.

In our study, we will employ only the continuous part of the nonlinear spectrum, that is, our data are encoded on and retrieved from the quantity *r*(*ξ*). The evolution of *r*(*ξ*) in the noise-free NLSE channel is trivial: 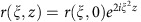, so that the orthogonality of nonlinear normal modes is preserved during the evolution. The inverse NFT (INFT) maps the encoded scattering data Λ at the transmitter onto the field *q*(*t*); see Fig. 1. This is achieved via the solution of Gelfand–Levitan–Marchenko equations^13^,^14^,^21^,^22^. Then, after the propagation over a fibre, at the receiver one reads the waveform *q*(*t*,*L*) and retrieves the nonlinear spectrum *r*(*ξ*;*L*) by solving the Zakharov–Shabat problem (2), that is, by the forward NFT. Unwinding the accumulated phase rotation inside the nonlinear domain, we finally recover the initial data, and this completes the NIS scheme (Fig. 1). Further details about the basics of the NIS can be found in refs 21, 22, 23 as well as in Supplementary Note 4.

### NFT data evolution in the presence of AWGN

The first goal of our study is to formulate the stochastic model for the data evolution inside the NFT domain. When the noise is small compared with signal power (the exact conditions are given in the Methods section), one can apply the inverse scattering transform perturbation theory^34^,^35^, which yields a self-consistent stochastic channel description inside the NFT domain. Namely, the dynamics of the continuous nonlinear spectrum is given by a linear equation with additive noise:


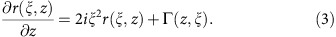


The nonlinear spectral noise Γ(*z*,*ξ*) is still a zero mean complex Gaussian process, but it possesses several properties distinguishing it from its space-time domain progenitor *η*(*z*,*t*). It is fully characterized by two complex autocorrelation functions: 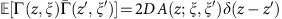 and 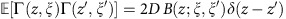. The explicit form of the functions *A* and *B* is determined by the projection of *η* on the nonlinear normal modes and is given in the Supplementary Note 2.

Thus, the signal evolution inside the nonlinear spectral domain amounts to the dispersive phase rotation affected by noise. One can note the similarities between the linear Fourier channel and its nonlinear counterpart (equation (3)): to see this, we can drop nonlinearity in equation (1) and rewrite it in the linear frequency domain as: *∂q*(*ω*,*z*)/*∂z*=−(*iω*^2^/2) *q*(*ω*,*z*)+*η*_*ω*_(*z*), where the noise, as in equation (1), has zero mean and the only nonzero autocorrelation function 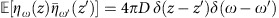. This similarity becomes even more striking if we recall that in the limit of low power the following relation between the FT and NFT spectra holds^14^,^33^: 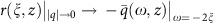. Applying this transformation to equation (3), we indeed recover circular AWGN with the linear power spectral density (PSD) 2*D*. However, the seeming simplicity of the evolution inside the NFT channel is deceptive. First, the new noise Γ is no longer circular, in contrast to its linear counterpart *η*_*ω*_(*z*). Next, this noise is neither homogeneous nor uncorrelated, as *A* and *B* are generally functions of both ‘frequencies' *ξ* and *ξ*′. The most important distinctive property of Γ, however, is that it depends on the initial spectrum, *r*(*ξ*,0). From the information theory perspective, the latter means that equation (3) defines an input-dependent Gaussian channel with memory^41^.

### The continuous NFT channel model

For the information-theoretic analysis, the channel model given by a stochastic equation (3) must be reformulated as an input–output probabilistic model, that is, the conditional probability density function (PDF) of the channel output given the channel input. We define the continuous channel output *Y*_*ξ*_ as the solution of equation (3) at the receiver located at distance *z*=*L* with the compensated phase rotation and filtering:


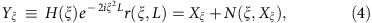


where *X*_*ξ*_≡ *r*(*ξ*,0), *H*(*ξ*) is the rectangular bandpass filtering function in the nonlinear frequency domain (applied at the receiver), that selects only a given channel of interest (COI). The effective filtered noise *N*(*ξ*,*X*_*ξ*_) (with zero mean) has the following correlation properties:


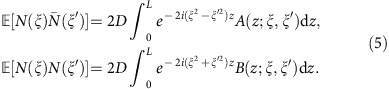


Naturally, due to the filtering the above relations hold within the COI only. We do not include any add/drop elements and optical–electrical conversion in our considerations here. But the possibility of including such elements and the lack of side information regarding them from the point of view of COI (which is a commonplace situation) makes the interference from other channels being effectively random, and in all our further calculations, we only consider a single (central) COI, and reckon the encoded information in other (than COI) channels as an additional contribution to the noise PSD (Supplementary Note 6). To evaluate autocorrelation functions (5), one needs to know the full *z*-evolution of the unperturbed Jost functions Φ(*z*;*t*,*ξ*), and this problem does not have a closed form solution in the general case. However, in the regime of a long fibre system one can either use the large *z* asymptotic solutions of Zakharov–Shabat problem (2)^43^,^44^, or the assumption of a finite temporal extent of the pulse, which is always the case for the NIS in a burst mode^19^,^23^ (Supplementary Note 3). Then, assuming large *L*, one obtains a remarkably simple result that explicitly depends only on the initial spectral data:


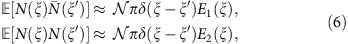


where *E*_1_(*ξ*)≡1+|*X*_*ξ*_|^2^+|*X*_*ξ*_|^4^ is an effective PSD (normalized to its linear value), 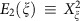, 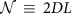. The latter quantity is the accumulated noise variance per sample in the time domain, and we have omitted the non-diagonal terms of order unity as small compared with those ∼*L* (Supplementary Note 3).

Two important observations regarding the properties of the noise *N*(*ξ*) can be made. First, within the nonlinear bandwidth of the COI, the noise PSD in the NFT domain, *E*_1_(*ξ*), grows nonlinearly with the spectral power of the input. Second, the channel model (4), (6) is local in the nonlinear frequency *ξ*. So, for example, in the case of dense WDM, one can simply match the nonlinear bandwidth of the filter with that of the COI and prevent both direct and noise-induced channel crosstalks without losing any of the informational content of our message, since the signal-dependent nonlinear spectral broadening is virtually absent. It is this remarkable property of the nonlinear spectrum (which holds as long as the effective signal-to-noise ratio (SNR_eff_) defined by equation (11) below is large and the propagation distance is not too small) that makes the NIS-based transmission potentially free from the crosstalk and bandwidth-related sources of the capacity degradation that plague most of the conventional transmission systems^3^,^4^,^5^. In the Methods section, we elaborate this statement further considering practical time-sampled multi-channels and in the Supplementary Note 6, we verify the PSD results above by a direct numerical simulation.

### Sampling and the discrete input–output model

So far we have defined our channel (4)–(6) using continuous field representation. The advantage of such an approach is that it allowed us to consider the multitude of the conventional schemes within the same theoretical framework. However, in digital communications, the signal is modulated and sampled in the time domain, and for each time sample, the information is encoded via complex amplitude level sets corresponding to discrete or continuous constellations^5^. Therefore, to make our results pertinent to the recently proposed NFT communication systems^21^,^22^,^23^, we shall consider two closely related standard frequency multiplexed schemes, namely, dense Nyquist WDM^5^ and OFDM^45^,^46^ both adapted to the NIS scheme.

We start from a general encoded sequence in time domain:


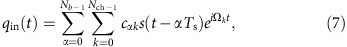


where *N*_b_ is the length of the symbol sequence (that is, burst), *N*_ch_ is the number of WDM channels or alternatively OFDM subcarriers, *s*(*t*) is the base wave-shape defining the particular format, *T*_s_, is the symbol width, Ω_*k*_ is an individual channel/subcarrier frequency. Here unless otherwise specified, we use normalized units. It is the discrete set of coefficients *c*_*αk*_ that now bears our informational content, and real and imaginary parts of *c*_*αk*_ form the components of the 2*M*-dimensional input X, with *M*=*N*_b_ × *N*_ch_. Within the NIS scheme, Fig. 1, we do not actually synthesize the waveform (7). Instead, we use its linear spectrum and use it as the nonlinear spectrum of a new optical signal *q*(*z*=0,*t*) to be launched into the fibre, utilising the mapping rule between the initial Fourier spectrum and the NFT reflection coefficient: 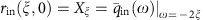. Note that the correlation properties of the nonlinear noise, (4)–(6), now explicitly depend on the amplitudes of the input sequence (7). The actual optical signal in the time domain is generated by applying the INFT. The resulting waveform is then fed into the optical fibre model (1). At large values of SNR_eff_, as defined by equation (11), the nonlinear spectrum evolves according to equation (3), and the input–output interrelation is then given by equations (4) and (5), assuming that at the receiver one uses NFT (2) to obtain the nonlinear spectrum, compensates for the propagation-accumulated phase and then retrieves the modulation coefficients of each symbol, *c*_*αk*_, using standard linear demodulation schemes^22^,^23^ (Supplementary Note 4). For the WDM case, the received nonlinear spectrum, *r*(*ξ*,*L*), is bandpass filtered for a given COI, see equation (4), while for the OFDM, the filtering is assumed over the total signal bandwidth. Note that the quantity 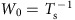 serves as the single channel bandwidth for the Nyquist WDM and carrier spacing for the OFDM case.

Since the channel in the nonlinear frequency domain (4)–(5) is characterized by additive Gaussian input-dependent noise (that is, the channel law inside the NFT domain is Gaussian with the input-dependent covariance), the discrete channel in the NIS scheme has the same property:


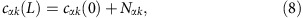


where *N*_*αk*_ is the projection of the spectral noise *N*(*ξ*,*X*_*ξ*_) onto the corresponding subcarrier in the OFDM case and the Nyquist-sampled noise vectors for the COI in the WDM case. Introducing 2*M* real and imaginary parts of *c*_*αk*_ as discrete real-valued input and output, **X** and **Y** correspondingly, one gets for the input–output conditional PDF the multivariate Gaussian distribution with the 2*M* × 2*M* quadrature correlation matrix 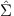 whose elements are obtained from the correlation functions (5). Since the intensities *A* and *B* from (5) depend on **X** (that is, on *r*(0,*ξ*)), so does the correlation matrix: 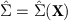. Using input (7) and asymptotic expressions (6), one obtains for the components of 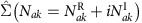:


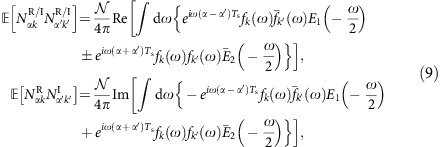


where *E*_1,2_(*ξ*) is defined below equation (6). Coefficients *f*_*k*_(*ω*) are the format-dependent form factors closely related to the linear Fourier transform of the pulse form *s*(*t*) from (7), see Supplementary Note 6. In the WDM case, this form factor is cut off by filtering and is only nonzero when the frequency belongs to the COI of width 2*π*/*T*_s_. Since the channels do not overlap, the noise components from different channels are uncorrelated. This is the consequence of the already mentioned property of the asymptotic absence of the channel crosstalk in the continuous model (4), (6). For the OFDM, the integration is restricted to the total nonlinear bandwidth of 2*πN*_ch_/*T*_s_.

### Capacity per symbol estimates for WDM/OFDM NIS transmission

For an arbitrary vector, information channel the input–output mutual information *I*(**X**,**Y**) is defined as^5^,^40^,^41^:


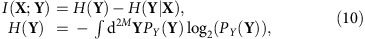


where *H* designates the entropy. The Shannon capacity per symbol, *C*, is the maximum of *I*(**X**;**Y**)/*M* over the input distribution *P*_*X*_(**X**) subject to the average power per sample constraint 
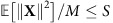
. For any additive Gaussian channel, the expression for the channel entropy *H*(**Y**|**X**) is obtained by averaging the determinant of the conditional correlation matrix **Σ**(**X**) over the input distribution. Our channel (8) possesses the non-diagonal input-dependent correlation matrix (9) that makes the direct optimization of the mutual information functional extremely difficult and only some lower bound for the channel capacity can be obtained. This is a common situation in case when the physical signal propagation is a nonlinear dynamical process. A standard approach for the lower-bound estimate is to use Gaussian input **X**_G_ with independently distributed real quadrature samples each having the variance *S*/2, which in the continuous limit corresponds to a Gaussian process with constant spectral density proportional to *S* (ref. 5). Another popular choice is the so-called ring constellation input,^4^,^5^ where for each complex sample the amplitude is fixed while the phase is uniformly distributed. Here we shall use the Gaussian input unless otherwise specified.

Analytical expressions for the mutual information for the channel given by equations (8)–(9), 24 are generally intractable even with the Gaussian independent identically distributed (i.i.d.) input. This is because of the forbiddingly complex dependence of the noise correlation matrix (9) on the input. Further standard step to achieve a tractable analytical result at the expense of the accuracy of the estimate is to use the effective Gaussian input–output model and the Pinsker's formula, keeping in mind that this bound may not be tight at all^39^. Despite that, this procedure is rather standard and its further details are outlined in the Methods section. There it is shown that in the limit of large effective SNR defined by the equation below, the Pinsker lower bound for the capacity, *C*_G_, in bits per symbol is found to be (real-world units are assumed)


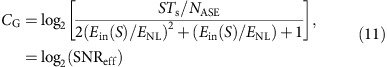


where the second line is the definition of the effective SNR, *N*_ASE_=*hν*_0_*K*_T_*χL* is the PSD of the accumulated ASE noise (see Supplementary Note 1 and refs 4, 5 for the physical meaning of each parameter), *E*_in_(*S*)=*ST*_s_*N*_b_*N*_ch_ is the average energy of the effective initial optical burst (before the NIS module); if one wants to express equation (11) in terms of *S* instead of *E*_in_, this can be done by means of this linear dependence given above. The quantity *E*_NL_=|*β*_2_|*N*_ch_/(*γT*_s_) represents a typical energy scale where the nonlinear effects become pronounced. This formula holds for both OFDM and Nyquist-based NIS transmission, and it is the main result of our paper. It is accurate up to terms of order *O*[1/SNR_eff_]; the general applicability criteria are discussed in the Methods section. Note that, for a fixed propagation distance *L* and symbol rate, it displays a characteristic peaky behaviour (that is, reaches a local maximum) in both average input power, *S*, and burst energy, *E*_in_, which is common to many Gaussian-based lower-bound estimates for conventional transmission formats^3^,^4^,^5^,^37^.

### Implications for long-haul optical systems

We can now put the obtained results into perspective by considering model of the fibre–optical communication systems operating on long-haul distances. For a fixed distance, the number of channels/subcarriers, burst duration and symbol rate the argument of log in (11) is a monotonically growing function of *E*_in_ (or *S*) up to 
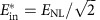
, which corresponds to the maximum of the estimated bound:


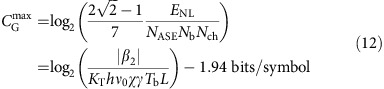


where *T*_b_=*T*_s_*N*_b_ is the duration of the burst before the NIS module, and the signal bandwidth is 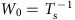. From equation (12), it is seen that the estimate deteriorates slowly (logarithmically) with the product *L* × *T*_b_. On the other hand, it does not depend on the number of channels, *N*_ch_, which is a direct consequence of the absence of the channel crosstalk.

Let us now address the physical meaning of equation (12), in particular, explain why a shorter burst duration brings about a higher capacity than a longer one. One can note that the denominator of equation (11) (which is actually the effective noise power in the NFT domain) grows with the ratio of the burst energy and the nonlinear energy. In other words, unlike the linear situation where the size of the burst does not affect the spectral properties of the noise, here this density grows both with the burst size and the energy of the pulse. The first circumstance is due to the fact that the noise in the nonlinear spectrum depends on the signal in time domain in the nonlocal manner: it depends on the integral characteristics of the time domain pulse rather than local ones (pretty much as the usual linear Fourier transform has the spectrum that depends on the whole time domain evolution of the pulse). The longer the burst duration *T*_b_, the more nonlinear noise it accumulates as it is dragged along—hence the capacity decreases. Now let us turn to the dependence of 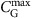 on the fibre parameters. By decreasing *β*_2_ and increasing the nonlinearity parameter *γ*, we effectively increase the dispersion length *L*_*D*_, while decreasing the nonlinear length *L*_NL_=1/(*γP*). In other words, one makes the system more nonlinear and lowers the energy threshold *E*_NL_ where the nonlinear effects are important. This, in turn, makes the aforementioned noise accumulation in the NFT domain more pronounced, such that its nonlinear spectral density grows, which has the adverse effect on SNR. This accumulation effect is clearly seen in the direct numerical simulation given in Supplementary Fig. 1 and Supplementary Note 6.

**Figure 2:**
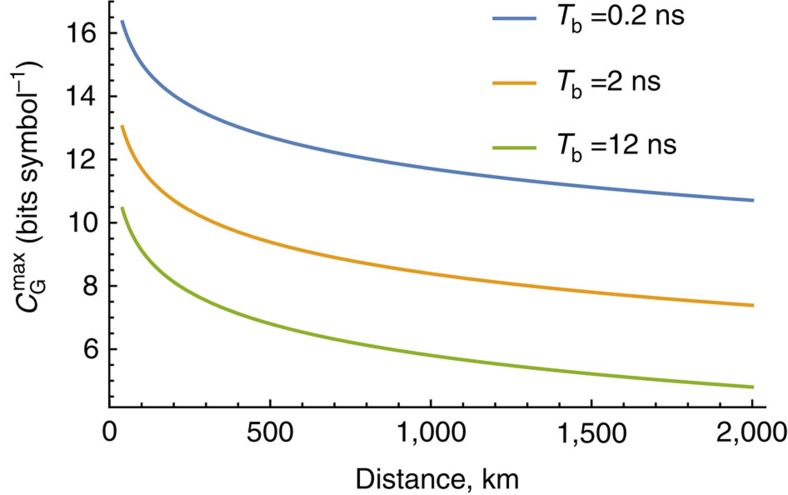
Lower bound for the maximum achievable capacity. Data obtained via equation (12) through the analysis of the approximate model (8), (9), versus transmission distance over 500 GHz bandwidth at different burst sizes.

Let us provide two typical examples of Nyquist and OFDM system parameters close to those reported both in conventional-4,5 and NIS-based^21^,^22^,^23^ systems. The goal here is not to compete with the record state-of-the-art experiments, but rather to give an idea of the required power levels and achievable rates. For the Nyquist transmission, we pick five channels with individual bandwidth 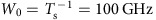, corresponding to the overall bandwidth of 500 GHz and for the OFDM we pick 100 subcarriers with the spacing of *W*_0_=5 GHz and the same effective total bandwidth. The optimal initial energy is then 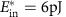 for both Nyquist and OFDM cases. The required power levels in the optical domain can be estimated by specifying the burst size, *N*_b_ (Supplementary Note 7). For the considered parameters, the lower bound on the capacity per symbol can be estimated as ∼10.7 bits per symbol over 500 GHz bandwidth at 2,000 km. Figure 2 plots the estimate (12) as a function of distance for different burst sizes. The result deteriorates with the burst size for both Nyquist and OFDM transmission as predicted by (12). Note however that for a fixed symbol rate the penalty of going from, say, 1,000 to 2,000 km is only 1 bit per symbol, so ∼1,000 km mark at least 12 bit per symbol can be achieved and so on. For the Nyquist case, varying the burst size is equivalent to changing the number of symbols in the burst while keeping the bandwidth fixed, whereas for the OFDM, one needs to change the number of subcarriers to keep the bandwidth fixed.

## Discussion

We have developed a theoretical approach based on the perturbation theory for the NFT data for the estimate of the lower bound of the capacity per symbol for the NFT-based optical transmission, which becomes asymptotically exact in the limit of large effective SNR. Considering transmission over 500 GHz bandwidth, the lower bound on the capacity per symbol is estimated as ∼10.7 bit per symbol at 2,000 km. The accurate estimates of the spectral efficiency corresponding to the capacity per symbol require massive system optimization in terms of achievable symbol rates. The NFT technique is still in the emerging early stages and the accurate estimates of the spectral efficiency corresponding to the capacity per symbol found in this paper would require the massive system optimization in terms of achievable symbol rates. In particular, one will need to obtain an explicit dependence of the linear frequency and time domain dependence of the pulse width and bandwidth on the system parameters. This seems to us to be a difficult task to achieve analytically and will require the full numerical optimization of various NFT systems that is well beyond the scope of a single paper. But some preliminary results and considerations can already be found in the Methods section and Supplementary Note 7. However, we would like to stress that the estimates of the capacity per symbol made in our work show great promise of the NFT technique and give an important guidance for the development of future systems. Moreover, some of the results presented here have high self-sufficient value. For example, equation (3) describes a continuous channel model for a generic NFT-based system in the presence of inline noise, while equations (4), (5) and (9) develop it further, introducing a discrete time channel model for the NFT-based transmission dealing with the continuous spectrum. The model predicts the absence of the nonlinear spectral broadening and channel crosstalk that makes it applicable to multi-user routed optical transmission systems. The developed channel models can be applied for the transmission system design, optimization and digital signal processing. Note also that in the Methods section, we also present the pioneering results for the simulations of the nonlinear spectral domain WDM transmission, addressing the issue of the crosstalk between the channels and revealing the absence of the latter in the considered NFT systems. Finally, we believe that the capacity estimate (11) is too conservative and can be improved. Indeed, our channel model with memory (8) is very close to a recently studied simpler model^47^, where it was shown that by a proper coding one can achieve a non-decreasing lower bound for capacity. In fact this result can be proven rigorously for any static, memoryless, power constrained communication channel^48^.

When the paper was under the review, a very recent ArXiv publication^49^ came to our attention. It reports simulation results for the normal dispersion NFT channel. Interestingly, the capacity rates shown there are close to those predicted in our paper for a slightly more relevant to the long-haul transmission case of the anomalous dispersion. This further supports our belief that the NFT-based methods are important tool for overcoming the capacity crunch.

## Methods

### A lower bound of the capacity of the nonlinear channel

To derive a lower bound using the mutual information (10) with the 2*M*-dimensional Gaussian input **X**_G_, here we largely follow a standard information theory approach, see, for example, ref. 3. Namely, we replace the channel output **Y** with another Gaussian, **Y**_G_ such that the joint Gaussian input–output PDF *P*_G_(**X**_G_,**Y**_G_) has the same binary correlation function as the original distribution *P*(**X**_G_,**Y**). This effective Gaussian channel provides yet another lower bound for the capacity and has one important advantage that its capacity, *C*_G_, can be calculated directly via the so-called Pinsker formula^36^,^39^:


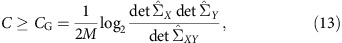


where 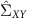 is the full input–output correlation matrix, while 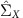 and 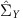 are input–input and output–output covariance matrices. The result is verified by the direct substitution of the multivariate Gaussian PDF *P*(**X**_G_,**Y**_G_) into the mutual information functional (10).

The fact that the conditional probability of the output is Gaussian simplifies the calculation of the determinant. Moreover, in Supplementary Note 5, it is shown that when SNR_eff_, defined in equation (11), is large, equation (13) simplifies to


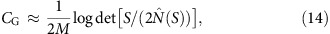


where the effective noise matrix 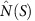 is the 2*M* × 2*M* correlation matrix (9) averaged over the i.i.d. Gaussian input with the variance *S*/2. It is the characteristic value of the noise matrix that defines the effective SNR in the problem and controls the validity of the model. To calculate it explicitly, one has to specify a particular transmission format. This is done in some detail in Supplementary Note 6, for the OFDM and Nyquist modulation of the input waveform (7). The result reads


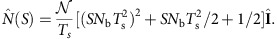


Plugging this result for the average noise matrix 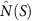 into the asymptotic estimate (14) and going back to the dimensional variables as discussed in the Supplementary Note 1, one obtains equation (11).

### The applicability of the obtained results

In the limit of small power and short burst, when *E*_in_<<*E*_NL_, the definition of the SNR_eff_ coincides with the linear one. However, in the nonlinear regime, the effective SNR deteriorates if one considers either a high-power regime or a very long burst. The overall consistency criteria for combining perturbation theory and asymptotic analysis can be written as SNR_eff_>>1, so that the validity condition for equation (3) is met, and the propagation distance *L* must be much greater than the dispersion length (defined via the total transmission bandwidth *W*=*N*_ch_*W*_0_), to assume the diagonal form of the correlation functions (6) that was further used to get equation (9). For the fixed fibre and burst parameters considered in the text, and the given symbol length *T*_s_, assuming the optimal energy 
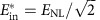
 corresponding to the optimal variance level 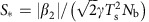, all the above conditions turn into a restricted window of distances in the real-world units:


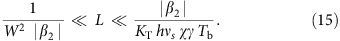


For *W*_0_=100 GHz and 5 WDM channels with the total bandwidth *W*=5*W*_0_=0.5 THz, and the burst size *T*_b_=12 ns in a standard telecom fibre, the above reads as 0.2<<*L*(km)<<2.1 × 10^5^, and this condition is easily met in all realistic implementations. Now let us study what are the theoretical restrictions on the input parameter *S*. In the nonlinear regime, when *E*_in_≳*E*_NL_, the quadratic term in the denominator of SNR_eff_ in equation (11) dominates and the condition SNR_eff_>>1 is equivalent to the following restriction:


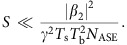


Thus, for the example considered above, that is, for the Nyquist modulation with five channels each having the bandwidth 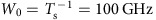, *T*_b_=12 ns and *L*=2,000 km considered in the text, the perturbative approach is valid up to the optical power levels *S*_opt_∼11 dBm.

At this point, we would like to explicitly clarify and explain some details of our results obtained. First, one has to keep in mind that in spite of our referring to the estimates as ‘lower bounds', our results are not an exact bound (in a rigorous mathematical sense) for the NLSE channel model: we presented the lower bound to an approximation of an initial model given by equations (8) and (9). Then, we have not considered full multiple-input–multiple-output system capacity and do not actually include add-drop multiplexers in the link believing, following ref. 5, that the case of COI with no side information corresponds to the worst case scenario capacity wise. We note that different assumptions for the input statistics can affect the capacity estimates^50^. We are assuming here that all channels are transmitting symbols with the same statistics and input power that is known at the receiver. According to the recently proposed classification of work^50^, this corresponds to the so-called adaptive interferer distribution. To avoid a possible confusion, one should notice that the result given by equation (11) does not conflict with the lower-bound estimation for the zero-dispersion channel obtained in ref 39: when the dispersion *β*_2_ goes to 0, the window of applicability of the result (11), given by two formulas above, closes, such that one cannot perform a correct comparison.

Another theory aspect is that, strictly speaking, the integrability of the NLSE (1) is lost due to the noise action (again, in a mathematical sense) even when one resides within the applicability limits protocolled above. However, when the conditions above are met, the NFT-type analysis still describes the system behaviour correctly, as it is guaranteed by the perturbation theory^34^,^35^. On the other hand, the perturbation theory used here cannot describe non-adiabatic phenomena like, for example, the creation of new solitonic eigenstates. Supplementary Fig. 4 from the Supplementary Note 7 demonstrates that there is no significant noise influence on the NFT domain bandwidth within the theory applicability range. Hoverer, there is still lack of the detailed study of how the signal bandwidth within the NFT domain behaves in response to the noise action when one is far beyond the perturbative regime.

### Evidence of the absence of the nonlinear channel crosstalk

In this section, we provide the results of the numerical simulations corroborating the predictions of the theory elaborated in the Results section above. One of the main challenges of the NFT method is to demonstrate that it is free from the nonlinear inter-channel interference that is thought to be the capacity bottleneck in the conventional systems.

**Figure 3:**
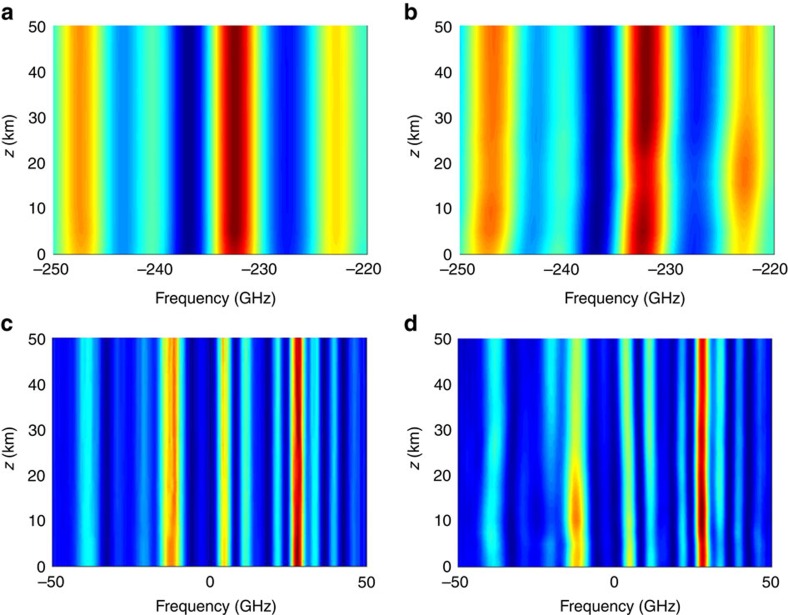
Linear versus Nonlinear Spectrum. (**a**) The evolution of the nonlinear spectrum of a 100 OFDM subcarriers with the 5 GHZ carrier spacing. (**b**) The evolution of the same sequence without the NIS module. (**c**) The evolution of the nonlinear spectrum for *N*_ch_=5 WDM channels in the NIS-based transmission. (**d**) The same but without the NIS block.

We aim at studying how both linear and nonlinear spectra evolve with the propagation distance. Since in the NIS scheme the nonlinear spectrum at the encoder and decoder coincides with the linear spectrum of the generated and detected sequences correspondingly, we shall present the results for the latter spectra in the real-world units. To be specific, we consider two cases: for the first, one we take 100 subcarriers OFDM over 500 GHz, and for the second example, we use the five-channel Nyquist-based WDM modulation with the same rate, as it was considered in the main text. For each format, in all our simulations, we used the same fixed realization of the symbol coefficients *c*_*αk*_ in the input form (7). As an example, we utilized the quadrature phase shift keying modulation of coefficients throughout. In all the cases, we compare the propagation in the NIS scheme with the same one in the absence of the NIS blocks, that is, when the signal (7) is actually synthesized and launched directly into the fibre (without digital NFT pre- and post-processing). To achieve a fair comparison, we made sure that the average optical power of the pulse launched into the fibre was the same both with and without NIS, so that *S*_opt_∼12 dBm for OFDM and ∼18 dBm for the Nyqust case. For the OFDM case, the input power levels were chosen to correspond to the optimal launch energy 
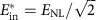
. For the Nyquist case, the power levels were chosen to be higher, to illustrate the stability of the NIS-based transmission. Note that in both cases the average signal level drops very quickly with the propagation distance due to the dispersion broadening, which in the absence of the soliton component decays almost as fast as it does in the linear case. Therefore, the nonlinear interaction between the different frequencies (which is expected to plague the conventional transmission, see the right column in Fig. 3) only takes place during the initial stages of evolution, and so there is no need to consider long spans. Also, due to the dispersion-induced power degradation the PSD of the signal very quickly becomes comparable with that of the noise making the spectral evolution curves uninformative. Therefore, in this section, we only present the spectral traces for the noise-free case, as the main goal here is to illustrate that the nonlinear spectra are immune to the nonlinear cross- and self-phase modulation. The results for the noise PSD under the same initial conditions are presented in Supplementary Note 6. The pulse evolution was studied by means of standard split-step scheme^1^ with a spatial step of 200 m. The forward and inverse NFT operations required for simulating the spectra in the left column were obtained using transfer matrix and Toeplitz matrix inversion respectively (see, for example, ref. 22).

In both cases, we are only showing the magnified part of the spectrum. For the OFDM, the higher four subcarriers are shown while for the Nyqusit case only the central COI is plotted. From Fig. 3, one can clearly see that the linear Fourier spectrum gets distorted during the conventional transmission (the right column), while its nonlinear counterpart remains robust (the left column).

Additionally in the Supplementary Note 6, we show how the nonlinear PSD evolves during the propagation in the multichannel environment similar to that of Fig. 3.

Together, with the simulations described above, we conducted a set of numerical experiments aimed at studying whether the soliton modes can emerge in the NIS scheme due to the noise action. Decreasing the effective correlation lengths of the numerical noise (the *z* interval of the noise injection during the NLSE simulations and the elementary time sample duration), we found that the amount of the total energy contained into the soliton degrees of freedom became <2% at 1,500 km when the time correlation duration was 5.25 ps and *z* correlation length 500 m, for a single channel with *W*_0_=100 GHz, *S*=22.3 dBm (32 Nyquist pulses in the burst). As we observed a steady tendency for the decrease of solitonic signal part with the contraction of correlation lengths in both time and space, we believe that, within the applicability limits of the perturbation theory, for the ‘very white' noise, the effect of solitonic constituents emerging from noise is of higher smallness order and can be neglected (at least, for the ideal Raman amplification case) within the leading order of perturbation approach developed in our work.

### Data availability

Data used to generate Figs 2 and 3 in this study are available in ‘Aston Research Explorer' portal with the identifier http://dx.doi.org/10.17036/73b24625-65c7-4ad5-bd35-26938c1e08e0. Additional data (including those used in the Supplementary Information) are available from the corresponding author on request.

## Additional information

**How to cite this article:** Derevyanko, S. A. *et al*. Capacity estimates for optical transmission based on the nonlinear Fourier transform. *Nat. Commun.* 7:12710 doi: 10.1038/ncomms12710 (2016).

## Supplementary Material

Supplementary Information Supplementary Figures 1-4, Supplementary Notes 1-7 and Supplementary References
