# Supplementary material for: Capacity estimates for optical transmission based on the nonlinear Fourier transform
Source: Nat Commun. 2016 Sep 9;7:12710. doi: 10.1038/ncomms12710 (PMC5023964; doi:10.1038/ncomms12710)
Supplement: Supplementary Information — Supplementary Figures 1-4, Supplementary Notes 1-7 and Supplementary References [file ncomms12710-s1.pdf]

Supplementary Information

Supplementary Figures

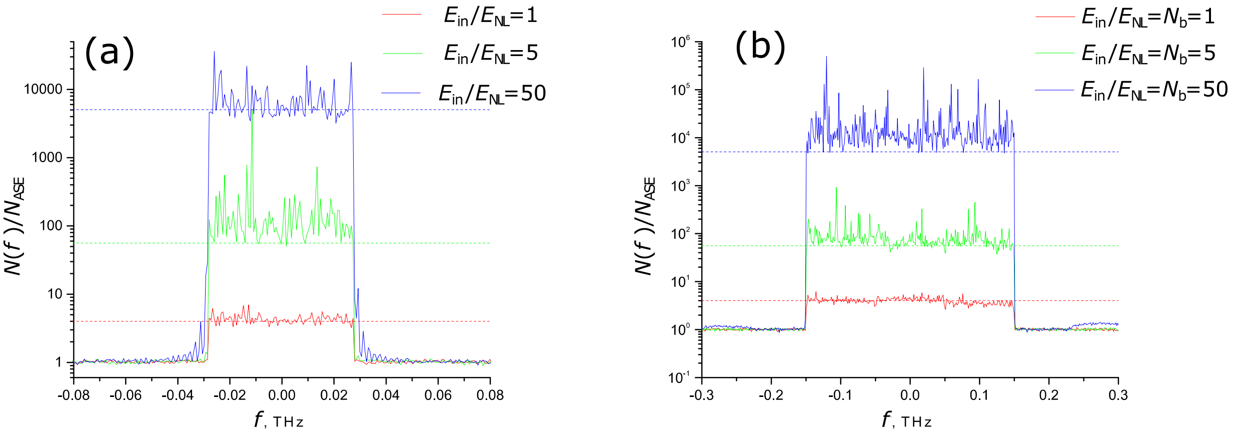

Supplementary Figure 1. **The simulated PSD of the nonlinear noise** (normalized to its linear counterpart N_{ASE}) for different input burst energies for OFDM (a) and Nyquist (b) sequences. Theoretical predictions of Eq. (38) are shown as dotted horizontal lines.

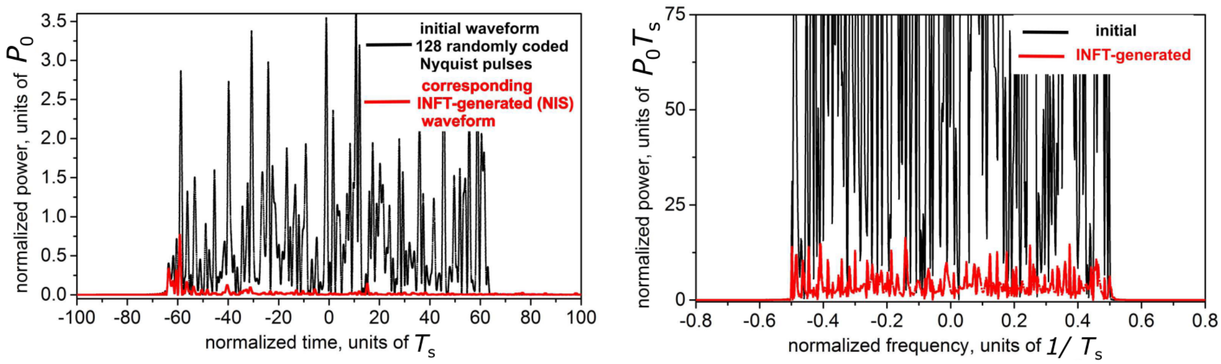

Supplementary Figure 2. **Time domain vs. linear spectrum of NIS-modulated sequences.** Left panel: Initial input burst consisting of 128 Nyquist pulses with the Gaussian distribution of coefficients with the variance being equal to $P_0/\sqrt{2}$ (normalized value) corresponding to the maximum spectral efficiency, see the main text. Right panel: Comparison of the linear Fourier spectra for both pulses from the left panel (normalized units).

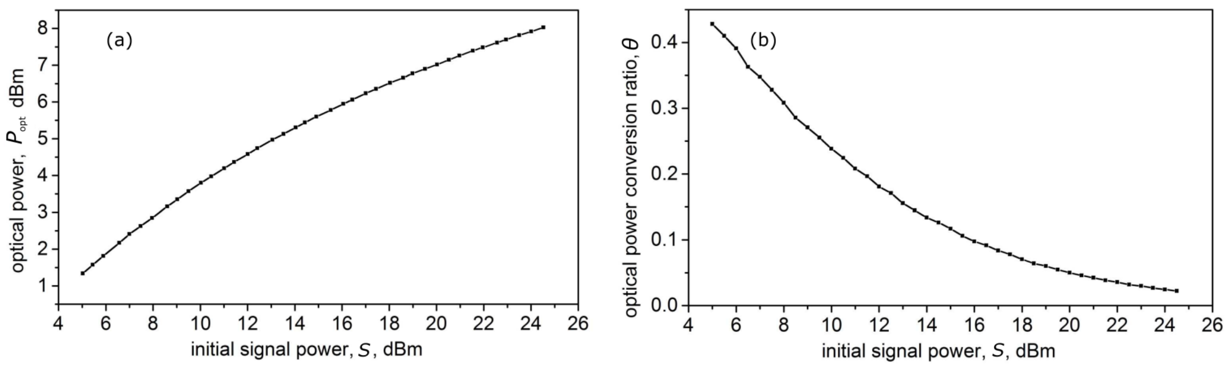

Supplementary Figure 3. **Power conversion between the optical and NFT domain** (a): Dependence of the average optical power P_{opt} in the optical domain on the average power of the channel input, S, for a burst $q_{\mathrm{in}}(t)$ containing 128 encoded Nyquist pulses with the Gaussian distribution of coefficients. (b): The averaged power conversion coefficient $\theta(S)$ as a function of the average initial signal power. Each point on the plots is the result of the averaging over 200 realizations.

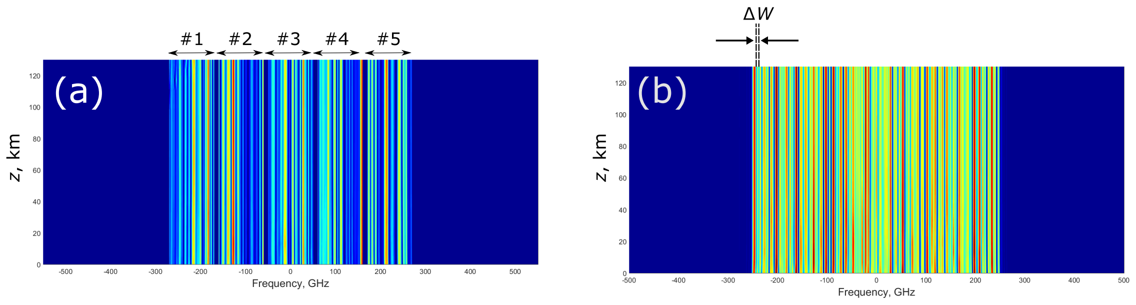

Supplementary Figure 4. **The evolution of the** ***linear spectrum*** **of the NIS encoded optical pulse in the optical fibre**. (a): Nyquist transmission (channels are indicated by arrows), (b): OFDM (subcarrier spacing of 5GHz is shown to guide the eye). The initial energy E_{in} is chosen at the optimal level $E_{\mathrm{NL}}/\sqrt{2}$.

Supplementary Note 1. NLSE AND THE DIMENSIONLESS UNITS

We consider as the master model the NLSE in the real world units with constant (anomalous) dispersion, describing the propagation of a complex slow-varying optical field envelope $Q(Z,\tau)$ inside a single-mode nonlinear fibre [1–3]:

$$\frac{\partial Q}{\partial Z} + i\frac{\beta_2}{2}\frac{\partial^2 Q}{\partial \tau^2} - i\gamma\, Q|Q|^2 = \tilde{\eta}(Z,\tau), \tag{1}$$

where Z stands for the propagation distance along the fibre and τ is the time in the frame co-moving with the velocity of the envelope. In this study we deal with the anomalous dispersion regime, assuming that the dispersion coefficient is negative, $\beta_2 < 0$: This situation corresponds to the so-called *focusing* type of the NLSE [1–3]; we use the typical value for silica fibres $\beta_2 \approx -22$ ps^2/km. The instantaneous Kerr nonlinearity coefficient γ characterizes nonlinearity strength, with the typical value $\gamma = 1.27$ (W·km)$^{-1}$. The model Eq. (1) can be applied either to path-averaging description of the practical fibre-optic systems with periodic gain and loss, or to the system with ideal Raman amplification (see for more details [1–3]). Here, for simplicity, but without loss of generality, we will refer to the latter case [3–5]. The AWGN term $\tilde{\eta}(Z, \tau)$ has the same correlation properties as the normalized quantity $\eta(z, t)$ from the main text (i.e. it is delta-correlated with circular polarization), aside from the explicit form of the autocorrelation intensity $\tilde{D}$ (noise power spectral density) [3] $2\tilde{D} = h\,\nu_0\,K_\mathrm{T}\,\chi$ where χ is the fibre loss coefficient, typically $\chi \approx 0.2$ dB/km at the carrying wavelength $\lambda_0 = 1.55$ μm (or $\chi = 4.61 \cdot 10^{-2}$ km^{-1}), K_T is the temperature-dependent factor (related to the phonon-occupancy factor) that characterizes the Raman pump providing the distributed gain; for the fibre-optic communication systems in normal conditions K_T is close to unity, $K_\mathrm{T} = 1.1$ to 1.2; ν_0 is the carrying frequency of the signal corresponding to λ_0: $\nu_0 = 193.55$ THz. Taking these typical values of parameters, one estimates the order of characteristic noise intensity per complex signal component, per unit of propagation length and per unit of bandwidth, to be $\tilde{D} \sim 10^{-21}$ J·km^{-1}; for $K_\mathrm{T} = 1.13$ we have: $\tilde{D} = 3.3 \cdot 10^{-21}$ J·km^{-1}.

In the paper body we use the explicit form of NFT operations attributed to the the normalized version of NLSE, Eq. (1). In our paper we normalize time in Eq. (1) to some characteristic time related to an input signal T_0. Normally this time is chosen to be equal to the symbol length, T_s, which is the reciprocal of the linear single channel bandwidth W_0 in the Nyquist case or carrier spacing in the OFDM case. Here, however, we prefer keeping this scale arbitrary to show the explicit independence of the obtained results from the chosen normalization. The distance is normalized by the following scale, $Z_0 = T_0^2/|\beta_2|$, which should not be confused with the dispersion length determined by the overall bandwidth of the system, $L_\mathrm{D} = 1/(W^2|\beta_2|)$. Finally, we measure the power of the field in units of $P_0 = (\gamma\, Z_0)^{-1}$ and normalize the signal amplitude correspondingly. The summary of normalizations is as

follows

$$\frac{\tau}{T_0} \to t, \qquad \frac{Z}{Z_0} \to z, \qquad \frac{\omega T_0}{2\pi} \to \omega, \qquad \frac{Q}{\sqrt{P_0}} = Q\,\sqrt{\gamma\, Z_0} \to q\,. \tag{2}$$

Thus Eq. (1) is recast it to the form (1) from the main text. When proceeding to normalized time, distance and power, the normalized noise intensity D scales as

$$D = \tilde{D}\,\frac{Z_0}{P_0\,T_0}. \tag{3}$$

The scaling of the dimensionless linear and nonlinear Fourier spectra follows from the above rules. For example the dimensionless linear noise PSD $\mathcal{N} = 2D(L/Z_0)$ is related to its dimensional counterpart $N_{\text{ASE}} = 2\tilde{D}L$ via

$$\mathcal{N} = \frac{N_{\text{ASE}}}{P_0\,T_0} = N_{\text{ASE}}\,\frac{\gamma T_0}{|\beta_2|}. \tag{4}$$

Supplementary Note 2. STOCHASTIC PERTURBATION THEORY FOR THE CONTINUOUS NONLINEAR SPECTRUM OF NLSE

The goal of this Note is to explain the derivation of the main continuous channel model – Eqs. (3) of the paper, as well as provide the general expressions for the correlations of the additive NFT noise $\Gamma(\xi, z)$. In this Note we assume the normalized quantities, as explained in Section Supplementary Note 1. However, at the beginning we note that a nice introduction and detailed description of the NFT method (often termed the inverse scattering technique), and related material, are amply presented in the initial work [6] and in the number of the excellent monographs on the subject, see, e.g., the book of Ablowitz and Segur [7]. The more general approach and alternative (equivalent) formulations of the scattering data sets (nonlinear spectrum) is presented in [8]. A lot of reference material and particular NFT details pertaining to the transmission based on the continuous spectrum is contained in the paper of current authors [9] (where, actually, the modulation of the continuous nonlinear spectrum part was first introduced). In [9] the relation between the NFT spectral data and the Fourier spectra are explicitly demonstrated. The perturbation theory for the NFT data [10, 11], considered further in this Note, operates with the projection of the (weak) perturbation onto the nonlinear normal modes, i.e. onto the continuous NFT modes in the case considered in our study.

While in the noiseless channel the evolution of the continuous spectral data amounts just to a simple phase rotation, the evolution of the perturbed system is generally unknown, which should come as no surprise as the perturbed NLSE is generally non-integrable. However, if the perturbation (inline noise) is small compared to the leading approximation (signal itself), one can use the well-known results of perturbation theory for the spectral data as given in Refs. [10, 11]; see also the applicability requirements in the Methods section of the paper body. According to this theory, when the system is close to integrable, the evolution of the continuous part of spectrum is given by the following equation:

$$\frac{\partial r}{\partial z} = 2i\,\xi^2\, r(\xi, z) - \frac{1}{a^2(\xi)}\, I[\mathbf{\Phi}(\xi, t; z), \mathbf{\Phi}(\xi, t; z)], \tag{5}$$

where $I[\mathbf{\Phi}(\xi, t; z), \mathbf{\Phi}(\xi, t; z)]$ is the projection of the noisy perturbation $\eta(z, t)$ on the corresponding unperturbed squared Jost functions. This operator, acting on two (arbitrary) vector functions $u = [u_1, u_2]$ and $v = [v_1, v_2]$, is defined by the expression

$$I[u, v] = \int_{-\infty}^{\infty} dt\, \Big[\eta(t, z)\, u_2(\xi, t, z)\, v_2(\xi, t, z) + \bar{\eta}(t, z)\, u_1(\xi, t, z)\, v_1(\xi, t, z)\Big]. \tag{6}$$

In Eq. (6) $a(\xi)$ is the Jost scattering coefficient [see the explicit definition of $a(\xi)$ in the main text paragraph after the Zakharov-Shabat problem introduction, Eq. (2)], and $\mathbf{\Phi} = [\phi_1, \phi_2]^T$ is the Jost function, i.e. a special solution of the Zakharov-Shabat problem, Eq. (2) of the main text, fixed by the conditions at the left infinity $\mathbf{\Phi} \xrightarrow{t \to -\infty} [e^{-i\xi t}, 0]^T$ (see the main text, the paragraph following Eq. (2)). The more detailed derivation of the explicit from of the projection (6) entering Eq. (5) can be found in the original works of Kaup and Newell [10, 11].

Since our treatment is a perturbative one, it is sufficient to evaluate $\mathbf{\Phi}(\xi, t; z)$, entering Eqs. (5), (6), in the leading zeroth order, i.e., for the noise-free system, inasmuch as Eq. (6) already contains a small parameter via the noise term η. Let us assume that we have *a full information* about the noiseless channel for a given input nonlinear spectrum, that is to say we know the Jost functions $\mathbf{\Phi}(\xi, t; z)$ and Jost coefficients $a(\xi)$ and $b(\xi; z)$ of the unperturbed *deterministic system* for arbitrary z. Then the quantity

$$\Gamma(\xi, z) = -\frac{1}{a^2(\xi)} \int_{-\infty}^{\infty} dt\, \left[\eta(t, z)\, \phi_2^2(\xi, t; z) + \bar{\eta}(t, z)\, \phi_1^2(\xi, t; z)\right] \tag{7}$$

is the renormalized additive Gaussian noise that now depends on the spectral parameter ξ rather than time variable t. Again, in the above formula all Jost functions and Jost coefficients should be understood as those of the unperturbed problem. Using the fact that $\eta(t,z)$ is delta-correlated circular Gaussian noise while the Jost function $\mathbf{\Phi}$ are deterministic, we get for the correlation functions, Eq. (5) of the main paper, with the spectral noise amplitudes, A and B, explicitly depending on the evolving Jost functions of the unperturbed problem (and, thus, on the initial distribution $r(\xi,0)$):

$$
\begin{aligned}
A(z;\xi,\xi') &= \frac{1}{a^2(\xi)\,\bar{a}^2(\xi')}\int\limits_{-\infty}^{\infty} dt\,\left[\phi_2^2(\xi,t;z)\,\bar{\phi}_2^2(\xi',t;z)+\phi_1^2(\xi,t;z)\,\bar{\phi}_1^2(\xi',t;z)\right],\\
B(z;\xi,\xi') &= \frac{1}{a^2(\xi)\,a^2(\xi')}\int\limits_{-\infty}^{\infty} dt\,\left[\phi_2^2(\xi,t;z)\,\phi_1^2(\xi',t;z)+\phi_2^2(\xi',t;z)\,\phi_1^2(\xi,t;z)\right].
\end{aligned}
\tag{8}
$$

Supplementary Note 3. SIMPLIFIED EXPRESSION FOR THE FAR FIELD CORRELATION FUNCTIONS OF THE NONLINEAR SPECTRAL NOISE $N(\xi)$

This Note is dedicated to the derivation of the simplified diagonal expression of the nonlinear spectral noise correlation functions – Eq. (6) of the main paper.

Finite extent inputs

Let us consider the case where the pulse has a finite extent: $q(t) \equiv 0$ if $t < T_1$ and $t > T_2$, with arbitrary function $q(t)$ inside the finite interval $[T_1, T_2]$ (we will omit z-dependence index for brevity). This is particularly relevant for the OFDM transmission where the envelope wave form has a rectangular shape. For the realistic NIS transmission the pulse is usually truncated to have a finite duration, and is padded by zero "wings" to suppress the inter-symbol (inter-burst) interference [12], such that the signals have an effectively finite duration during the whole evolution. It is convenient to split the integrals in the general expression for the amplitudes Eq. (8) into tree subintervals, $t < T_1$, $T_1 < t < T_2$, and $t > T_2$. (Such an assumption complies, again, with the "burst mode" modulation requirement for the NFT-based systems, that has to be used for the NIS to avoid inter-symbol interference, see more explanations in Section Supplementary Note 7 below and Refs. [12, 13].) The quantities

referring to the first interval, i.e. to the left from the $q(t)$ extent, will be marked with the superscript "$<$"; the quantities for the interior interval where $q(t) \neq 0$ will be indexed with the superscript "T", and the remaining interval to the right from the $q(t)$ extent is marked with "$>$". Further, we will need the Fourier transform (FT) of the Heaviside unit step function, $H(t) = 1$ for $t > 0$ and 0 otherwise:

$$H(\omega) = \int_{-\infty}^{\infty} dt\, H(t)\, e^{-i\omega t} = -\frac{i}{\omega} + \pi\,\delta(\omega), \tag{9}$$

where the first term is to be understood in the sense of principal value.

For the first interval to the left from the potential, $t < T_1$, we easily obtain from the Zakharov-Shabat problem (Eq. (2) of the main paper):

$$\phi_1^{<}(\xi, t) = e^{-i\xi t}, \qquad \phi_2^{<}(\xi, t) = 0. \tag{10}$$

By the definition of the right Jost functions, for the interval from the right side, $t > T_2$, we have

$$\phi_1^{>}(\xi, t) = a(\xi)\, e^{-i\xi t}, \qquad \phi_2^{>}(\xi, t) = b(\xi)\, e^{i\xi t}. \tag{11}$$

Now one can calculate the partial contributions to the correlation functions at $z = 0$. We start from the region to the left from the $q(t)$ extent and, substituting Eq. (10) into (8), one gets:

$$A^{<}(\xi, \xi') = \frac{\pi}{2}\,\frac{1}{|a(\xi)|^4}\,\delta(\xi - \xi') + \frac{i}{2}\,\frac{1}{a^2(\xi)\bar{a}^2(\xi')}\,\frac{e^{-2i(\xi-\xi')T_1}}{\xi - \xi'}, \tag{12}$$

and $B^{<}(\xi, \xi') = 0$. The next contribution stems from $\phi_i^{>}(\xi, t)$. In a similar fashion, using the expression for the Fourier transform of the Heaviside function (9) we obtain

$$A^{>}(\xi, \xi') = \frac{\pi}{2}\left(1 + |r(\xi)|^4\right)\delta(\xi - \xi') + \frac{i}{2}\,\frac{1}{\xi - \xi'}\left(r^2(\xi)\bar{r}^2(\xi')\, e^{2i(\xi-\xi')T_2} - e^{-2i(\xi-\xi')T_2}\right), \tag{13}$$

$$B^{>}(\xi, \xi') = \pi\, r^2(\xi)\,\delta(\xi - \xi') + \frac{i}{2}\,\frac{1}{\xi - \xi'}\left(r^2(\xi)\, e^{2i(\xi-\xi')T_2} - r^2(\xi')\, e^{-2i(\xi-\xi')T_2}\right). \tag{14}$$

In the linear limit, $r \to 0$, function B disappears altogether, while A retains only δ-contribution, so we have a correct transition to the linear FT-domain autocorrelation functions intensities, see the subsection "NFT data evolution in the presence of AWGN" of the main paper text. Both correlation functions have an essential singularity at $\xi = \xi'$. Additionally, there is the contribution to (8) from the area inside the pulse extent $T_1 < t < T_2$:

$A^T(\xi,\xi')$ and $B^T(\xi,\xi')$, which can be obtained if one substitutes the finite integration limits $T_{1,2}$ in place of the infinite ones in Eq. (8). However, these terms are generally non-singular and, as we shall see below, at large propagation distances L their contribution can be neglected.

The total correlation function values are the sum of the partial contributions:

$$A = A^{<} + A^T + A^{>}, \qquad B = B^T + B^{>}. \tag{15}$$

Assuming for simplicity the symmetric endpoints of the signal extent, $T_{1,2} = \mp T/2$, and inserting z-dependence of r and b in (15), $r(\xi;z)$, $b(\xi;z) \propto e^{2i\xi^2 z}$, we arrive at the following answer (neglecting insignificant A^T and B^T contributions):

$$\begin{aligned} A(\xi,\xi';z) = \frac{\pi}{2}\,\frac{1}{|a(\xi)|^4}\left(1+|a(\xi)|^4+|b(\xi)|^4\right)\delta(\xi-\xi') + \frac{i}{2}\,\frac{1}{\xi-\xi'}\,\frac{1}{a^2(\xi)\,\bar{a}^2(\xi')}\times \\ \left[e^{i(\xi-\xi')T} - b^2(\xi)\,\bar{b}^2(\xi')\,e^{i(\xi-\xi')T}\,e^{4iz(\xi^2-\xi'^2)} + a^2(\xi)\,\bar{a}^2(\xi')\,e^{-i(\xi-\xi')T}\right], \end{aligned} \tag{16}$$

$$B(\xi,\xi';z) = \pi\, r^2(\xi)\,e^{4iz\xi^2}\,\delta(\xi-\xi') + \frac{i}{2}\,\frac{1}{\xi-\xi'}\left[r^2(\xi)\,e^{i(\xi-\xi')T}\,e^{4iz\xi^2} - r^2(\xi')\,e^{-i(\xi-\xi')T}\,e^{4iz\xi'^2}\right]. \tag{17}$$

We note that for large propagation distances, $z = L \gg L_{\mathrm{D}}$, the main contribution to the noise correlation functions (5) of the main paper is $O[L]$ and comes from the diagonal terms in A and B, proportional to $\delta(\xi-\xi')$. One can easily check that the nondiagonal terms have oscillating z-dependence which after integration gives the contribution $O[1]$; the same applies to the intermediate region $[T_1, T_2]$, giving the contributions from terms A^T and B^T being in the order of 1. With this in mind, it is sufficient to keep only the diagonal part of the correlation functions A and B, Eqs. (16), (17). Then, Eq. (6) of the main paper follows after the trivial z-integration, where one also uses an identity relating absolute values of Jost coefficients [7, 8]: $|a(\xi)|^2 = (1+|r(\xi)|^2)^{-1}$.

Far field approximation of Jost functions

The equation (5) of the main paper can be also derived by using the large distance asymptotes for the Jost functions first obtained by Zakharov and Manakov [14] (see also [7]). Their asymptotic analysis split the far field area of the pulse into the resonant area in the vicinity of the point $t = -2z\xi$ and the asymptotically free areas far from the resonant

point. In the latter region one has a particularly simple expression for the Jost function: for $t \ll -2z\xi$ we have

$$\mathbf{\Phi}(\xi,t;z) = \begin{pmatrix} e^{-i\xi t} \\ 0 \end{pmatrix} \exp\left[\frac{i}{2} \int\limits_{-t/2z}^{\infty} \frac{\alpha^2(\lambda)}{\lambda-\xi}\, d\lambda \right], \tag{18}$$

and for $t \gg -2z\xi$

$$\mathbf{\Phi}(\xi,t;z) = \begin{pmatrix} a(\xi)\, e^{-i\xi t} \exp\left[-\frac{i}{2}\int_{-\infty}^{-t/(2z)} \frac{\alpha^2(\lambda)}{\lambda-\xi}\, d\lambda \right] \\ b(\xi)\, e^{2i\xi^2 z + i\xi t} \exp\left[\frac{i}{2}\int_{-\infty}^{-t/(2z)} \frac{\alpha^2(\lambda)}{\lambda-\xi}\, d\lambda \right] \end{pmatrix}, \tag{19}$$

where $\alpha^2(\lambda) \equiv \pi^{-1} \log|a(\lambda)|^{-2}$ and $1 \ll z \le L$. As before, the Jost coefficients $a(\xi)$ and $b(\xi)$ denote their initial values at $z = 0$. In the resonant area the phases of the Jost functions experience large fluctuations [7, 14] and hence this area produces no significant contribution to the amplitudes (8).

To simplify Eqs. (18), (19) even further, note that the linear spectral magnitude $|r(\xi)|^2$ of a multi-symbol burst can be represented as fast fluctuations around the bandwidth-averaged value $|\bar{r}|$ (the overbar here and in the formula below identifies averaging). The scale of these fast fluctuations is $\tilde{B}/N_{\mathrm{b}}$, where N_{b} is the number of symbols in the burst and $\tilde{B} = 2\pi W$ is the nonlinear (circular) bandwidth of the initial pulse sequence. Since the function $\alpha^2(\lambda) = \log(1 + |r(\lambda)|^2)$ has the same behaviour one can replace $\alpha^2(\lambda)$ from (18), (19), when substituting it in the integrals (8) by its average value $\overline{\alpha^2}$ and cutoff the infinite integration limits by the nonlinear bandwidth window $|\lambda| < \tilde{B}/4$. With this simplifications one can write

$$\mathbf{\Phi}(\xi,t;z) \approx \begin{pmatrix} e^{-i\xi t + \frac{i}{2}\overline{\alpha^2}\log\left|\frac{\tilde{B}-4\xi}{2t/z+4\xi}\right|} \\ 0 \end{pmatrix} H[-t-2z\xi] + \begin{pmatrix} a(\xi) e^{-i\xi t - \frac{i}{2}\overline{\alpha^2}\log\left|\frac{2t/z+4\xi}{\tilde{B}+4\xi}\right|} \\ b(\xi)\, e^{2i\xi^2 z + i\xi t + \frac{i}{2}\overline{\alpha^2}\log\left|\frac{2t/z+4\xi}{\tilde{B}+4\xi}\right|} \end{pmatrix} H[t+2z\xi]. \tag{20}$$

Again, as in the previous subsection, for the large propagation distances $L \gg L_{\mathrm{D}}$ the main contribution to the noise correlation functions (5) of the main paper stems from the diagonal terms proportional to $\delta(\xi - \xi')$. This diagonal part is exactly identical to the diagonal part of (16) and (17), which were obtained with a different method, and plugging it into Eq. (5) of the main paper Eq. (6) follows straight away.

Supplementary Note 4. THE BASICS OF NIS AND GENERAL EXPRESSIONS FOR THE AUTOCORRELATION FUNCTIONS OF $N^{\mathrm{R,I}}_{\alpha,k}$

At the beginning of this Note we must mention that the flowchart explaining the nonlinear inverse synthesis (NIS) method idea with the exemplifying profiles after each processing stage is given in Fig. 1 of Ref. [15], together with detailed explanation of each method's step. The thorough assessment of the NIS performance in terms of the Q-factor behaviour in dependence on the input power for the distributed ideal Raman amplification case is given in [12] for the OFDM and Nyquist modulations of the nonlinear spectral data (or, rather, of the profile $q_{\mathrm{in}}(t)$ in the notations of our paper). The method generalization and the NIS performance for EFDA (lumped) and non-ideal realistic Raman amplification cases are considered in the recent publications [13] and [16], respectively.

In this Note we derive Eq. (9) of the main paper. We start from the auxiliary input waveform given by Eq. (7) of the main paper and, in accordance with the NIS scheme [15], perform a forward linear FT to obtain the linear spectrum (all in the units of Section Supplementary Note 1):

$$q_{\mathrm{in}}(\omega) = \sum_{\alpha=0}^{N_{\mathrm{b}}-1} \sum_{k=0}^{N_{\mathrm{ch}}-1} c_{\alpha k}\, \tilde{s}(\omega - \Omega_k)\, e^{i\,\omega\,\alpha\,T_{\mathrm{s}}}, \tag{21}$$

where $s(\omega)$ is the linear FT of the base wave-shape. Next, the crucial point for the NIS is to define the mapping between the linear spectrum (21) and the quantity $r(\xi)$ from the nonlinear spectral domain. The convenient choice (proposed in Refs. [12, 15]) is to keep the correspondence between the initial waveform, Eq. (7) of the main text, and the generated NIS waveform in the linear limit, i.e. when $|q(t)| \sim |q(\omega)| \to 0$. As we have the following relation between the nonlinear and linear spectra in the limit of low powers [7, 8, 15]:

$$r(\xi) \xrightarrow{|q|\to 0} -\bar{q}|_{\omega=-2\xi}\,, \tag{22}$$

it is convenient to map the linear spectrum (21) to the nonlinear spectrum $r(\xi,0)$ via the transformation [15]:

$$X_\xi = r(\xi,0) = -\sum_{\alpha=0}^{N_{\mathrm{b}}-1} \sum_{k=0}^{N_{\mathrm{ch}}-1} \bar{c}_{\alpha k}\, \bar{\tilde{s}}(-2\xi - \Omega_k)\, e^{2i\,\xi\,\alpha\,T_{\mathrm{s}}}. \tag{23}$$

After applying the INFT to the above nonlinear spectrum (23), the generated pulse is launched into the fibre channel and propagates towards the receiver (with the inline noise).

As discussed in Section Supplementary Note 7, the launched optical pulse will generally have different (smaller) energy of a burst as well as smaller average power level in the optical domain. At the detection stage a forward NFT is performed and after removing the accumulated phase rotation and filtering out all the WDM channels apart from the channel of interest (see Eq. (4) of the main paper) a backward *linear* FT is performed at the receiver ultimately producing a continuous signal input-output relation:

$$q(L,t) = q(0,t) - \frac{1}{2\pi}\int \bar{N}(-\omega/2)\, e^{i\,\omega\, t}\, d\omega, \tag{24}$$

where the noise N is exactly the same random process as featured from Eq. (5) of the main paper onwards and the integration is limited to the filter bandwidth.

The actual recovery of the output coefficients $c_{\alpha k}(L)$ depends on the modulation scheme. Let us start with the dense WDM transmission based on Nyquist pulses allowing one to achieve a hight spectral efficiency [17]. We note that this is the same scheme as used in e.g. Ref. [3] for *conventional* transmission scheme with digital back propagation and in [12] in the framework of NIS. This corresponds to choosing a sinc pulse, $s(t) = \sin \pi W_0 t/(\pi W_0 t)$, as the base wave-form in Eq. (7) of the main paper.

The detection scheme that we assume here consists of using pass band filtering matched to the exact bandwidth W_0 of channel of interest (COI) followed by sampling at symbol rate given by Nyquist-Shannon criterion: $T_\mathrm{s} = 1/W_0$.

Hence from (24) it follows that for the Nyquist transmission we have:

$$c_{\alpha k}(L) = c_{\alpha k}(0) - \frac{1}{2\pi}\int d\omega \bar{N}(-\omega/2)\, e^{i\omega\,\alpha\, T_\mathrm{s}}, \tag{25}$$

where the nonlinear spectral noise is filtered around COI k only, which provides the frequency cut-off in the integration. This equation is nothing else than Eq. (8) of the main text written here in the expanded form exactly for the Nyquist signal modulation.

The next format that we are going to consider is the popular orthogonal frequency division multiplexing format (OFDM) [18], since it was also discussed in detail in other NIS-related publications [12, 15]. Now the explicit form of of the carrier in Eq. (7) of the main text is $s(t) = \Pi(t/T_\mathrm{s})$, where $\Pi[x]$ is the rectangle function of unit height in the interval $[0, 1]$. One has the orthogonality inside the time domain [17, 18]

$$\delta_{kl} = \frac{1}{T_\mathrm{s}}\int_0^{T_\mathrm{s}} s(t)s(t)\, e^{i\,(\Omega_k - \Omega_l)\, t}\, dt, \tag{26}$$

which holds as long as the subcarrier frequencies satisfy $\Omega_k - \Omega_l = 2\pi m/T_\mathrm{s}$ with an integer m. The coefficients $c_{\alpha k}$ are then recovered by a FT of the signal:

$$c_{\alpha k}(L) = \frac{1}{T_\mathrm{s}} \int\limits_{\alpha T_\mathrm{s}}^{(\alpha+1)T_\mathrm{s}} q(L,t)\, e^{-i\,\Omega_k t}\, dt. \tag{27}$$

Here for simplicity we shall assume that the subcarriers are symmetrically placed around zero so that $\Omega_k = 2\pi(k - N_\mathrm{ch}/2)/T_\mathrm{s}$ and their number N_ch is even.

For the OFDM modulation the expression given by Eq. (8) of the main text can explicitly rewritten as

$$c_{\alpha k}(L) = c_{\alpha k}(0) - \frac{1}{2\pi} \int \bar{N}(-\omega/2)\, e^{i(\omega-\Omega_k)(\alpha+1/2)T_\mathrm{s}}\, \frac{\sin\left[(\omega-\Omega_k)T_\mathrm{s}/2\right]}{(\omega-\Omega_k)T_\mathrm{s}/2}\, d\omega. \tag{28}$$

The filtering in the OFDM case (unlike the WDM) is applied over the whole bandwidth occupied by multiple carriers.

From Eq. (4) of the main paper, using Eqs. (25), (28) one obtains general expressions for the components of the correlation matrix, applicable to any nonlinear frequency-multiplexed format:

$$\begin{aligned}
\mathbb{E}\left[N^\mathrm{R}_{\alpha k}\, N^\mathrm{R}_{\alpha' k'}\right] &= \frac{1}{8\pi^2}\,\mathrm{Re}\Bigg\{ \int\!\!\int d\omega\, d\omega' e^{i\omega\,\alpha\,T_\mathrm{s}}\, f_k(\omega) \\
&\times \Big(\mathbb{E}[\bar{N}(-\omega/2) N(-\omega'/2)]\, e^{-i\omega'\alpha'\,T_\mathrm{s}} \bar{f}_{k'}(\omega') + \mathbb{E}[\bar{N}(-\omega/2)\bar{N}(-\omega'/2)]\, e^{i\omega'\alpha'\,T_\mathrm{s}} f_{k'}(\omega') \Big) \Bigg\}, \\
\mathbb{E}\left[N^\mathrm{I}_{\alpha k}\, N^\mathrm{I}_{\alpha' k'}\right] &= \frac{1}{8\pi^2}\,\mathrm{Re}\Bigg\{ \int\!\!\int d\omega\, d\omega' e^{i\omega\,\alpha\,T_\mathrm{s}}\, f_k(\omega) \\
&\times \Big(\mathbb{E}[\bar{N}(-\omega/2) N(-\omega'/2)]\, e^{-i\omega'\alpha'\,T_\mathrm{s}} \bar{f}_{k'}(\omega') - \mathbb{E}[\bar{N}(-\omega/2)\bar{N}(-\omega'/2)]\, e^{i\omega'\alpha'\,T_\mathrm{s}} f_{k'}(\omega') \Big) \Bigg\}, \\
\mathbb{E}\left[N^\mathrm{R}_{\alpha k}\, N^\mathrm{I}_{\alpha' k'}\right] &= \frac{1}{8\pi^2}\,\mathrm{Im}\Bigg\{ \int\!\!\int d\omega\, d\omega' e^{i\omega\,\alpha\,T_\mathrm{s}}\, f_k(\omega) \\
&\times \Big(\mathbb{E}[\bar{N}(-\omega/2)\bar{N}(-\omega'/2)]\, e^{i\omega'\alpha'\,T_\mathrm{s}} f_{k'}(\omega') - \mathbb{E}[\bar{N}(-\omega/2) N(-\omega'/2)]\, e^{-i\omega'\alpha'\,T_\mathrm{s}} \bar{f}_{k'}(\omega') \Big) \Bigg\},
\end{aligned} \tag{29}$$

where the integration is assumed over the whole bandwidth of the multichannel signal, the correlation functions $\mathbb{E}[NN']$ and $\mathbb{E}[N\bar{N}']$ are taken from Eq. (5) of the main paper and the form-factor functions $f_k(\omega)$ for the aforementioned Nyquist and OFDM modulation formats

are explicitly written as

$$f_k(\omega) = \begin{cases} e^{i(\omega-\Omega_k)T_s/2}\,\frac{\sin[(\omega-\Omega_k)T_s/2]}{(\omega-\Omega_k)T_s/2} & \text{for OFDM modulation,} \\ P_k(\omega) & \text{for dense Nyquist modulation.} \end{cases} \tag{30}$$

Here $P_k(\omega)$ is the indicator function for the k-th WDM channel: it equals 1 when the frequency is inside the band and zero otherwise.

In the asymptotic regime, $L \gg L_{\mathrm{D}}$, since the noise correlation functions given by Eq. (6) of the main text are diagonal as shown in Section Supplementary Note 3, the double frequency integration in (29) reduces to a single Fourier transform of the corresponding nonlinear input-dependent spectral densities, so that Eq. (9) of the main text follows.

Supplementary Note 5. THE ASYMPTOTIC FORM OF THE PINSKER ESTIMATE (14) FOR LARGE SNR

According to Pinsker estimate for the capacity (13) of the main paper, one needs to calculate the determinant of the three correlation matrices. For the input correlations one has $\hat{\mathbf{\Sigma}}_X = (S/2)\,\mathbf{I}_{2M}$ (where $\hat{I}_{2M}$ is $2M \times 2M$ unitary matrix). Using the fact that the conditional output is Gaussian one has for the output correlation matrix $\hat{\mathbf{\Sigma}}_Y = \hat{\mathbf{\Sigma}}_X + \hat{\mathbf{N}}(S)$ where the components of $\hat{\mathbf{N}}(S)$ are determined from (29) by averaging over the i.i.d. Gaussian input X_G. As for the full $4M \times 4M$ input-output correlation matrix, it has the form:

$$\hat{\Sigma}_{XY} = (S/2)\hat{\mathbf{I}}_{4M} + \begin{pmatrix} 0 & 0 \\ 0 & \hat{\mathbf{N}}(S) \end{pmatrix}. \tag{31}$$

Then, in view of the above, the Pinsker bound for capacity can be written as

$$C_G = \frac{1}{2M}\,\log_2 \frac{\det\left[\hat{\mathbf{I}}_{2M} + 2\hat{\mathbf{N}}(S)/S\right]}{\det\begin{pmatrix} \hat{\mathbf{I}}_{2M} & \hat{\mathbf{I}}_{2M} \\ \hat{\mathbf{I}}_{2M} & \hat{\mathbf{I}}_{2M} + 2\hat{\mathbf{N}}(S)/S \end{pmatrix}}. \tag{32}$$

The determinant in the denominator can be calculated using a well known formula for the determinant of the 2×2 block matrices [19]:

$$\det\begin{pmatrix} \hat{\mathbf{A}} & \hat{\mathbf{B}} \\ \hat{\mathbf{C}} & \hat{\mathbf{D}} \end{pmatrix} = \det(\hat{\mathbf{A}} - \hat{\mathbf{B}}\hat{\mathbf{D}}^{-1}\hat{\mathbf{C}})\det\hat{\mathbf{D}}. \tag{33}$$

Applying this formula to the Pinsker determinant one obtains

$$C_G = -\frac{1}{2M} \log_2 \det \left[\hat{\mathbf{I}}_{2M} - (\hat{\mathbf{I}}_{2M} + 2\hat{\mathbf{N}}(S)/S)^{-1} \right]. \tag{34}$$

Let us next assume that the effective SNR, $S/N(S)$ is large (by $N(S)$ we denote a typical value of the $\hat{\mathbf{N}}$ matrix). Then after expanding (34) in powers of $\hat{\mathbf{N}}/S$, in the main order one obtains the following simple result:

$$C_G = \frac{1}{2M} \log_2 \det \left[S/(2\hat{\mathbf{N}}(S)) \right] + O[N(S)/S] \tag{35}$$

The above is Eq. (14) from the main paper.

Supplementary Note 6. INPUT-AVERAGED CORRELATION MATRIX $\hat{N}(S)$ FOR OFDM AND NYQUIST TRANSMISSION AND THE PSD OF THE NONLINEAR NOISE

According to Eq. (35), to get a lower bound for the channel capacity one must first average the noise-correlated matrix, Eq. (9) of the main paper, over i.i.d. circularly polarized input Gaussian symbols $c_{\alpha k}$ with the average power $\langle |c_{\alpha k}|^2 \rangle = S$. Here we perform the calculation for an arbitrary i.i.d. input subject to the above constraint. To this end one must start with the expression (23), plug it into generalized spectral densities E_1 and E_2 and average these over the coefficients $c_{\alpha k}$. The second order momenta do not present significant difficulties, in particular the non-circular part $\langle E_2 \rangle$ vanishes and the only non-trivial task is to calculate the 4-th order term $\mathbb{E}[|X_\xi|^4]$. It is easy to see that the only non-zero contribution comes from the quadruplets of all identical indexes as well as matched pairings (second order momenta) as can be induced from the Wick theorem in the Gaussian case. The final result reads

$$\begin{aligned} \langle E_1(-\omega/2) \rangle = 1 + S N_\mathrm{b} \sum_k |\tilde{s}(\omega - \Omega_k)|^2 + 2 S^2 N_\mathrm{b}^2 \left(\sum_k |\tilde{s}(\omega - \Omega_k)|^2 \right)^2 \\ + \left(\frac{\mathbb{E}[|c|^4]}{\mathbb{E}[|c|^2]^2} - 2 \right) S^2 N_\mathrm{b} \sum_k |\tilde{s}(\omega - \Omega_k)|^4. \end{aligned} \tag{36}$$

The term in the brackets in the second line depends on the statistics of the coefficients $c_{\alpha k}$ and vanishes in the circular Gaussian case. The expression above is particulary simple for the Nyquist case as within each channel the spectral function is constant, $\tilde{s}(\omega - \Omega_k) = T_\mathrm{s}$, and for each frequency only one channel at a time contributes to the sums over k. For the

OFDM the spectral function is again a re-scaled sinc with $|s_\omega(\omega)|^2 = \sin^2(\omega T_\mathrm{s}/2)/(\omega/2)^2$ and some extra work is required. Assuming that the number of subcarriers is large, $N_\mathrm{ch} \gg 1$, and the frequency is not too close to the band edges $\pm\pi N_\mathrm{ch}/T_\mathrm{s}$, one can approximately write for any positive integer p:

$$\sum_{k=0}^{N_\mathrm{ch}-1} |s_\omega(\omega-\Omega_k)|^{2p} \approx \frac{T_\mathrm{s}}{2\pi}\int\limits_{-\pi N_\mathrm{ch}/T_\mathrm{s}}^{\pi N_\mathrm{ch}/T_\mathrm{s}} |s_\omega(\omega-\Omega)|^{2p} d\Omega \approx \frac{T_\mathrm{s}^{2p}}{2\pi}\int\limits_{-\infty}^{\infty} \frac{\sin^{2p} x/2}{(x/2)^{2p}}\, dx = T_\mathrm{s}^{2p}\, f(p), \quad (37)$$

where $f(1) = 1$ and $f(2) = 2/3$.

Thus in both cases the average power spectral density is flat inside the corresponding band and in the Gaussian case it can be written as

$$\langle E_1(-\omega/2)\rangle = 1 + SN_\mathrm{b}T_\mathrm{s}^2 + 2S^2N_\mathrm{b}^2T_\mathrm{s}^4. \quad (38)$$

Finally one must integrate the average density over the COI of width $2\pi W_0 = 2\pi/T_\mathrm{s}$ in the Nyquist case and over the OFDM band $[-\pi N_\mathrm{ch}/T_\mathrm{s}, \pi N_\mathrm{ch}/T_\mathrm{s}]$ for the OFDM case with the form factor function $f_k(\omega)$. In the OFDM one again can replace the finite limits of integration with the infinite ones and make use of the orthogonality of sinc subcarriers:

$$\int\limits_{-\infty}^{\infty} e^{i2\pi x(\alpha-\alpha')}\,\mathrm{sinc}[x-k]\,\mathrm{sinc}[x-k']dx = \delta_{kk'}\,\delta_{\alpha\alpha'}. \quad (39)$$

The final result for the input-averaged correlation matrix is the same for both OFDM and Nyquist and reads:

$$\mathbb{E}[N^\mathrm{R}_{\alpha k}N^\mathrm{I}_{\alpha' k'}] = 0, \quad \mathbb{E}[N^{R/I}_{\alpha k}N^{R/I}_{\alpha' k'}] = \frac{\mathcal{N}}{2T_\mathrm{s}}\left(1 + SN_\mathrm{b}T_\mathrm{s}^2 + 2S^2N_\mathrm{b}^2T_\mathrm{s}^4\right)\delta_{\alpha\alpha'}\,\delta_{kk'}. \quad (40)$$

Finally let us compare the theoretical predictions with the results of the numerical simulations for both OFDM and Nyquist systems. For both cases we chose the parameters close to the ones considered in the main text. We plot the numerically extracted ratio of the NFT noise PSD to its linear value N_ASE (i.e. the function E_1 from the main paper text) as a function of linear frequency for different values of the energy ratio $E_\mathrm{in}/E_\mathrm{NL} = (S/P_\mathrm{s})N_\mathrm{b}$ where $P_\mathrm{s} = |\beta_2|/(\gamma T_\mathrm{s}^2)$ is a typical nonlinear power in the r.w.u. In all the simulations the propagation distance L (and hence the linear PSD) was fixed at 500 km. We have used

$N_{\rm ch} = 3$ channels for Nyquist and $N_{\rm ch} = 112$ subcarriers for the OFDM. For the OFDM transmission the length of the burst was fixed, $N_{\rm b} = 1$, and we changed the energy ratio by simply increasing the power S. For the Nyqusit case we have fixed $S = P_{\rm s}$ and changed the number of symbols in the burst to achieve the desired energy ratio. The averaging was performed both over noise and over Gaussian-sampled set of the input symbols $c_{\alpha k}$.

From Fig. S1 one can see that the spectrum-averaged PSD confirms reasonably well with theoretical predictions as long as the energy is of the order of the nonlinear energy $E_{\rm in} \sim E_{\rm NL}$. As the energy is increased one gets a slight increase of the average level compared to Eq. (38) as well as occasional spikes. This occurs due to the two main reasons. Firstly, the error of the existing forward NFT solvers grows with the input signal power (see e.g. [12, 13]) even in the noise free environment. Secondly, the fluctuations on top of the average background can also partially occur due to the occasional parasitic soliton creation as discussed in the end of the Methods section from the paper body, which can significantly modulate the NFT spectrum of the signal and noise for a given realization. Note, however, that the optimal input energy values found in the main paper text are in the order of $E_{in} = E_{NL}/\sqrt{2} \sim E_{NL}$, where the agreement between theory and numerics is excellent for both OFDM and Nyqusit cases.

Supplementary Note 7. OPTICAL DOMAIN ESTIMATIONS

To end up, in this Section we present some additional details of the NIS method, Fig. 1 of the main paper (see also Fig. 1 of Refs. [12, 15] for more details and the block diagram of NIS-based optical communication systems, correspondingly), using mainly the Nyquist modulation for illustration purposes. We aim at looking at our transmission system in the space-time domain (although the transmission system itself is defined inside the nonlinear spectral domain). The physical aspects of the NIS approach were described in much detail in Ref. [15], and the performance of the method for OFDM and Nyquist NFT-based systems was thoroughly assessed in [12, 13].

According to the NIS method, at the transmitter one performs the one-to-one digital mapping of the input wave-shape linear spectrum, $q_{\rm in}(\omega)$, of the corresponding effective time domain signal $q_{\rm in}(t)$ (with average power S), onto the nonlinear spectrum of a new signal, see Eq. (23); our choice of mapping form, Eq. (22) (in the dimensionless units), ensures the

convergence to the linear spectrum in the low power limit. Then the new profile in the time domain, corresponding to initial $q_{\rm in}(\omega)$, is synthesized using the INFT [12, 15], i.e., by solving the Gelfand-Levitan-Marchenko equations [7, 8] starting from the corresponding profile of $r(\xi)$, given in our case by Eq. (23). We call this new synthesized waveform in the space-time domain the optical domain signal. For the high values of S this signal can be essentially different from the initial waveform $q_{\rm in}(t)$, see the examples in Ref. [15]. It is important that for the NIS transmission one must assume that our encoded signal is separated into "bursts", i.e. into the return-to-zero messages (in time domain) surrounded by zero guard intervals. This is stipulated by the fact that both NFT and INFT operations have to be performed with the finite-extent signals (see Fig. 4 of Ref. [12]). So, the NIS approach is appropriate for the burst mode transmission of a multi- or single-access network, in which neighbouring packets are separated by a guard interval. The guard time duration is usually chosen longer than the channel memory, i.e. the fibre chromatic dispersion induced memory, which precludes the inter-symbol (or rather "inter-burst") interference between neighbouring data packets. Different packet data can be sent from the same or different transmitters. Another important property of any NFT-based transmission method is that the energy in the optical domain is a nonlinear functions of $|r(\xi)|^2$, and hence of the average power S of the original modulated sequence, $q_{\rm in}(t)$. Indeed, the value of the total (dimensionless) energy of the optical domain signal can be expressed through the nonlinear spectrum via [7]:

$$E_{\rm norm} = \int_{-\infty}^{\infty} dt\, |q(t,z)|^2 = \frac{1}{\pi}\int_{-\infty}^{\infty} d\xi\, \ln|a(\xi)|^{-2} = \frac{1}{\pi}\int_{-\infty}^{\infty} d\xi\, \ln(1+|r(\xi)|^2), \tag{41}$$

where we have explicitly assumed the absence of soliton states and omitted the corresponding contribution to the energy. To get a real-world quantity one must multiply the above by P_0T_0. One can see that only in the linear limit, when the log can be expanded, does the optical domain energy scale linearly with power (the linear FT regime).

The interesting issue with regard to the NIS is how the signal characteristics change when one proceeds from the nonlinear spectral domain to the time domain. Fig. S2, left panel, shows the typical initial shape realization $q_{\rm in}(t)$ for the burst comprising 128 Nyquist orthogonal sinc profiles, where each coefficient is an independent identically distributed complex number picked from a Gaussian distribution, with zero average and variance equal to $P_0/\sqrt{2}$. Here one can evidently see how the energy conversion formula (41) works in practice, since, visually, the INFT-generated profile in optical domain has got much lower

power (energy) than the progenitor pulse $q_{\rm in}(t)$. Yet another property of the INFT generated pulses is the formation of an advancing tail (a very weak hardly visible one in our case) and effective broadening of the resulting pulse in the time domain: These effects were mentioned in Refs. [12, 13, 15], see Fig. 2 of the last reference. The next essential question is what happens to the linear bandwidth of the signal when one generates the NIS input: The right panel in Fig. S2 shows the linear Fourier spectra of two respective profiles given in the left subfigure. The important observation there is that the spectrum width for the whole power interval of interest remains almost the same as it is for the initial pulse $q_{\rm in}(\omega)$. This shows that the dispersion length estimation and the typical spectrum width given in the main text still remain in force when one deals with the NIS system in the optical domain and corresponding INFT-generated profiles evolving inside the optical fibre. The confirmation of the latter fact can also be found in the end of this Note.

Now let us address the question of the typical optical power and energy of the burst that are actually launched in the optical fibre in the NIS scheme. Assuming that the input is in the form (7) of the main paper and the coefficients are i.i.d. complex variables (not necessarily Gaussian) with the variance $\mathbb{E}[c_{\alpha k}\bar{c}_{\alpha' k'}] = S\,\delta_{kk'}\delta_{\alpha\alpha'}$, the average energy of an optical burst at the *input* of the NIS channel is given by

$$E_{\rm in} = \mathbb{E}\left[\int\limits_{-\infty}^{\infty} dt\,|q_{\rm in}(t)|^2\right] = S\,N_{\rm ch}\sum_{\alpha=0}^{N_{\rm b}-1}\int\limits_{-\infty}^{\infty}|s(t-\alpha T_{\rm s})|^2 = S\,T_{\rm s}\,N_{\rm ch}\,N_{\rm b}\,, \qquad (42)$$

and the result holds for both OFDM and Nyquist formats.

The expression above should not be confused with the actual energy of the generated pulse in the optical domain, $E_{\rm opt}$, that can be calculated using either the averaging of Eq. (41) or by the straightforward integration of the respective profiles in the time domain, see Fig. S2 for the example, followed by the averaging. Let us perform analytical calculation first. The input nonlinear spectrum $r(\xi) = X_\xi$ is given by Eq. (23). Then, we need to plug this expression in Eq. (42) and average the result over the i.i.d amplitudes. The technical problem of averaging a log function can be circumvented by means of central limit theorem. Indeed, at a large number of i.i.d. amplitudes one can assume that X_ξ is a Gaussian process with zero mean and flat average spectrum already calculated in the previous Note: $\mathbb{E}[|X_\xi|^2] \equiv \sigma^2 = SN_{\rm b}T_{\rm s}^2$. Then $|X_\xi|^2$ that enters an argument of the log function is exponentially distributed with the

mean σ^2. The average can be performed analytically yielding

$$\mathbb{E}[\log(1+|r(\xi)|^2)] = -e^{\sigma^{-2}}\,\mathrm{Ei}[-\sigma^{-2}], \tag{43}$$

where $\mathrm{Ei}[x]$ is an exponential integral. For long bursts, when $\sigma^2 \gtrsim 1$, one can expand this function and get a simpler formula $\mathbb{E}[\log(1+|r(\xi)|^2] \approx \log\sigma^2 - \gamma_E$, where $\gamma_E \approx 0.57$ is the Euler constant (it can be neglected in the main order). The further integration is restricted to half the total bandwidth (because $\xi = -\omega/2$). The resulting expression for the optical energy of the burst is then given by:

$$E_{\mathrm{opt}} = \frac{N_{\mathrm{ch}}}{T_{\mathrm{s}}}\left(\log[S\,N_{\mathrm{b}}\,T_{\mathrm{s}}^2] - \gamma_E\right), \quad S\,N_{\mathrm{b}}\,T_{\mathrm{s}}^2 \gtrsim 1. \tag{44}$$

Let us rewrite this important result in the real world units:

$$\begin{aligned} E_{\mathrm{opt}}[r.w.u.] = E_{\mathrm{NL}}\left|\mathrm{Ei}\left[-\frac{E_{\mathrm{NL}}}{E_{\mathrm{in}}}\right]\right| \exp\left[-\frac{E_{\mathrm{NL}}}{E_{\mathrm{in}}}\right] \approx E_{\mathrm{NL}}\left(\log\left[\frac{E_{\mathrm{in}}}{E_{\mathrm{NL}}}\right] - \gamma_E\right), \\ E_{\mathrm{in}} \gg E_{\mathrm{NL}} \equiv \frac{|\beta_2| N_{\mathrm{ch}}}{\gamma T_{\mathrm{s}}}, \end{aligned} \tag{45}$$

and in the low power regime, $E \ll E_{\mathrm{NL}}$, the log function can be expanded, so that $E_{\mathrm{opt}} \approx E_{\mathrm{in}}$. The above relation shows that the initial NIS block consisting of forward linear FT followed by the reciprocal NFT is a *lossy* procedure — the energy of the burst in the optical domain is logarithmically suppressed compared to the case when no NIS encoding is used and the modulated sequence (7) of the main paper is actually prepared and launched in the optical domain provided that the energy is greater than the nonlinear threshold E_{NL}. Of course if the NIS block implementation is purely digital, no actual energy conversion takes place as one generates directly the resulting optical signal.

The relation between the average optical domain power, $P_{\mathrm{opt}} = E_{\mathrm{opt}}/(T_{\mathrm{b}}N_{\mathrm{s}})$, and the average channel input power S is given in the left panel of Fig. S3, while the power conversion coefficient $\theta(S) = P_{\mathrm{opt}}(S)/S$ is shown in the right panel. Note that this coefficient is less than unity and goes down as S increases, which means that the higher is the power of the initial modulated sequence, the lesser fraction of it is actually transferred into the launched pulse in the optical domain, which is the consequence of the energy saturation discussed above.

Let us define the optical SNR (OSNR) in the r.w.u. as [3]:

$$\mathrm{OSNR}(S) = \frac{1}{2}\,\frac{P_{\mathrm{opt}}(S)}{N_{\mathrm{ASE}}\,W_{\mathrm{ref}}} = \frac{1}{2}\,\frac{S\,\theta(S)}{N_{\mathrm{ASE}}W_{\mathrm{ref}}}, \tag{46}$$

where $W_{\rm ref}$ is some reference bandwidths. Now, following [3], we define the "traditional" optical domain SNR as $\text{SNR} = 2\,\text{OSNR}\,\frac{W_{\rm ref}}{W}$, implying that our signal uses a single polarization state; according to the right panel of Fig. S2, the spectrum width of the INFT-generated signal for the range of initial signal powers $S \sim 5$ to 25 dBm is very close (almost identical) to the bandwidth W of the initial signal $q_{\rm in}(\omega)$); for simplicity we can also put $W_{\rm ref}/W \approx 1$. In reality we have to account for the need for the burst mode inasmuch as for the NFT-based transmission methods (not only for the NIS) one has to process the whole pulse since the NFT operations are attributed to vanishing (or truncated) signals. Because of this we arrive at the effective extension of the time domain for the NFT-based systems. Particularly, for the NIS scheme, Fig. 1 of the main paper, the burst generated in the time domain from a given encoded wave-shape contains a decaying tail, the extent of which depends on the initial pulse power (or, rather on its total energy) in a nonlinear way [12, 15], but the total generated pulse is always wider that its progenitor in the nonlinear spectral domain. Already the presence of the tail usually adds 20 to 50 percents to the initial pulse duration (the truncation of the tail may result in the error increase for the data transmitted). For the weakly-localized inputs, like the Nyquist modulation, the NIS-generated waveform is localized even worse and one has to process even bigger time-domain window. So, the larger extent of the pulse in the time domain reduces the effective SE of the NIS system by a factor of 1.5 to 2 and more. We would like to stress that most of the NFT studies currently deal with the vanishing boundary condition signals, or in the engineering terms, operation in the burst mode. This typically makes spectral efficiency of such transmission reduced compared to the case of a continuous signal processing. However, it is important to recognise that this a very early stage of the NFT applications in optical communications. The development of the analogue of the fast Fourier transform for the NFT system - fast NFT with the periodic boundary conditions [22, 23], will increase substantially spectral efficiency of the NFT while keeping higher robustness against nonlinear impairments.

Finally, let us study how the *linear* spectrum of NFT signal evolves during the propagation. We have seen form Fig. 3 of the main text that the individual nonlinear spectral features in the NIS-encoded pulse have not been degraded by fibre nonlinearity while the conventionally encoded pulse of the same energy has experienced distortions. It is only left to check that the linear bandwidth of the NIS-encoded pulse does not experience significant spectral broadening during the propagation so that the spectral efficiency at least

is not affected by the growth of the *linear* bandwidth of signal.) To this end in Fig. S4 we plot the linear spectral evolution, i.e. the linear spectra evolution corresponding to the NIS-encoded pulse. We have used the same parameters (burst duration, the number of subcarriers/channels) as in Fig. 3 of the main text. The only exception is that we have reduced the energy of the 20-symbol Nyquist burst to the optimal value $E_{\mathrm{NL}}/\sqrt{2}$ to make a uniform comparison to the OFDM case and introduced 10 GHz guard bands in the nonlinear spectrum. Note that here we are interested in the overall bandwidth changes and ignore the inside distortions of the linear spectrum (which seem to be much more pronounced in the OFDM case). One can see that in the selected interval the overall linear bandwidth increase is negligible in the first 100 km of propagation after which the dispersive broadening makes the system essentially linear and the effect is not expected to occur at all.

To sum up, even the burst mode of the NFT-based methods that is, certainly, not optimal in terms of the spectral efficiency, shows potential of the NFT technique in terms of capacity per symbol. Further development and optimization of NFT systems will improve their performance. The straightforward way is to incorporate the remaining discrete nonlinear spectrum degrees of freedom and combine the nonlinear frequency division multiplexing approach [20] with the NIS, for the sake of increasing the amount of information embedded into the same time and spectral volume (recall that in the absence of noise solitonic and radiation modes stay orthogonal inside the nonlinear spectral domain). The first example of such an advanced technique was recently proposed in [21]. Yet another way is to optimize further the time-domain occupation of the NIS-generated pulses using non-traditional formats tailored specially for the NFT-based transmission purposes, but this direction has yet to be developed. The most important direction is development of the periodic NFT-based processing [22]. Overall, we note that the effective absence of the inter-mode cross-talk inside the nonlinear domain and other results of our study demonstrate the great potential of the NFT application, where the main problem now lies in the optimization and further elaboration of the approach rather than in some inevitable performance degradation sources, such as e.g. channel crosstalk.

SUPPLEMENTARY REFERENCES

---

[1] G. P. Agrawal, *Fiber-Optic Communication Systems, 4th ed.* (Wiley-Blackwell, New Jersey, 2010).

[2] Iannoe, E., Matera, F. Mecozzi, A., & Settembre, M. *Nonlinear Optical Communication Networks*, (John Wiley & Sons, New York, 1998).

[3] R.-J. Essiambre, G. J. Foschini, G. Kramer, & P. J. Winzer, Phys. Rev. Lett., **101**, 163901 (2008); R. Essiambre, G. Kramer, P. J. Winzer, G. J. Foschini, & B. J. Goebel, J. Lightwave Technol. **28**, 662–701 (2010).

[4] J. D. Ania-Castanon, V. Karalekas, P. Harper, & S. K. Turitsyn, Simultaneous Spatial and Spectral Transparency in Ultralong Fiber Lasers, Phys. Rev. Lett. 101, 123903 (2008)

[5] J. D. Ania-Castanon, T. Ellingham, R. Ibbotson, X. Chen, L. Zhang, & S. K. Turitsyn, Phys. Rev. Lett. 96, 023902 (2006)

[6] V. E. Zakharov & A. B. Shabat, Soviet Physics-JETP **34**, 62–69 (1972).

[7] M. J. Ablowitz, & H. Segur, *Solitons and the Inverse Scattering Transform* (SIAM, Philadelphia, 1981).

[8] N. J. Ablowitz, D. J. Kaup, A. C. Newell, & H. Segur, Stud. Appl. Math. **53**, 249–315 (1974).

[9] J. E. Prilepsky, S. A. Derevyanko, & S. K. Turitsyn, Opt. Express **21**, 24344–24367 (2013).

[10] D. J. Kaup, SIAM J. Appl. Math. **31**, 121–133 (1976)

[11] D. J. Kaup & A. C. Newell, Proc. Roy. Soc. Lond. A Mat. **361**, 413–446 (1978).

[12] S. T. Le, J. E. Prilepsky, & S. K. Turitsyn, Opt. Express **22**, 26720–26741 (2014).

[13] S. T. Le, J. E. Prilepsky, & S. K. Turitsyn, Opt. Express **23**, 8317–8328 (2015).

[14] V. E. Zakharov & S. V. Manakov, Sov. Phys. JETP **44**, 106–112 (1976); for the higher order expansions see V. I. Novokshenov, Soviet Physics-Doklady, **251**, 799–802 (1980).

[15] J. E. Prilepsky, S .A. Derevyanko, K. J. Blow, I. Gabitov, & S. K. Turitsyn, Phys. Rev. Lett. **113**, 013901 (2014).

[16] S. T. Le, J. E. Prilepsky, M. Kamalian, P. Rosa, M. Tan, J. D. Ania-Castanon, P. Harper, & S. K. Turitsyn, "Optimized Nonlinear Inverse Synthesis for Optical Links with Distributed Raman Amplification," in 41st European Conference on Optical Communications (ECOC),

Valencia, Spain, paper Tu 1.1.3, 2015.

[17] R. Schmogrow, M. Winter, M. Meyer, D. Hillerkuss, S. Wolf, B. Baeuerle, A. Ludwig, B. Nebendahl, S. Ben-Ezra, J. Meyer, M. Dreschmann, M. Huebner, J. Becker, C. Koos, W. Freude, & J. Leuthold, Opt. Express **20**, 317–337 (2012).

[18] W. Shieh, H. Bao, & Y. Tang, Opt. Express **16**, 841–850 (2008).

[19] J. R. Silvester, Math. Gazette **84**, 460–467 (2000).

[20] Hari, S., Kschischang, F. & Yousefi, M. "Multi-eigenvalue communication via the nonlinear Fourier transform." In *27th Biennial Symposium on Communications (QBSC)*, Kingston, ON, Canada, pp. 92–95 (2014).

[21] I. Tavakkolnia & M. Safari, "Signalling over nonlinear fibre-optic channels by utilizing both solitonic and radiative spectra," in *IEEE European Conference on Networks and Communications (EuCNC)*, Paris, France, pp. 103-107 (2015).

[22] S. Wahls & H. V. Poor, IEEE Trans. Inf. Theory **61**, 6957–6974 (2015).

[23] M. Kamalian, J. E. Prilepsky, S. T. Le, and S. K. Turitsyn, "Optical communication based on the periodic nonlinear Fourier transform signal processing," *IEEE 6th International Conference On Photonics (ICP)*, Sarawak, Malaysia (2016).
